# Pressure ulcer practice in European hospitals: a scoping review

**DOI:** 10.1016/j.ijnsa.2025.100477

**Published:** 2025-12-23

**Authors:** Jan Kottner, Ulrike Linstedt, Ahmed Tafesh, Monira El Genedy-Kalyoncu

**Affiliations:** Charité-Universitätsmedizin Berlin, corporate member of Freie Universität Berlin and Humboldt-Universität zu Berlin, Germany

**Keywords:** Pressure ulcer, Prevention, Treatment, Hospital

## Abstract

**Background:**

Pressure ulcer prevention and management belong to the core topics of nursing practice and research for decades. Numerous systematic reviews, clinical practice guidelines, and good practice documents are available, but pressure ulcer prevention and treatment may not be conducted following state-of-the-art methods in clinical practice.

**Objectives:**

The overall objective was to describe and map the available evidence regarding pressure ulcer prevention and treatment practices in European hospitals.

**Methods:**

A scoping review was conducted in the databases Embase and Medline (via OVID) and CINAHL including descriptive empirical studies published between 2014 and 2024. The population included all healthcare professionals, and context was hospitals in the European Union.

**Results:**

Forty-six studies from 18 countries were included, describing 33 structure and 32 process aspects. The categories knowledge (*n* = 19) and attitudes (*n* = 11) were examined or identified by far the most frequently. Formal education and qualification were mentioned 10 times and personal interest and/or commitment 7 times. At the system level, the availability of and access to clear standards or protocols was mentioned most frequently. Most frequent process aspects included pressure ulcer risk assessment (*n* = 10), the type of risk assessment (*n* = 7), skin assessment (*n* = 10), repositioning (*n* = 9) and the use of support surfaces (*n* = 7).

**Conclusion:**

More than 60 organizational, structural, and process characteristics were identified as potentially influencing pressure ulcer practice in European hospitals. Individual knowledge and attitudes and the availability of standards/protocols were most often investigated indicating a perceived priority in shaping pressure ulcer practice. Since all studies indicate knowledge deficits and a lack of implementation of basic prophylactic measures, there seems to be high potential to improve pressure ulcer prevention and enhance patient safety.

**What is already known**

• Pressure ulcer prevalence and incidence are high in hospital settings.
• Pressure ulceration is one of the common sources of potentially preventable patient harm.
• Pressure ulcer prevention and treatment may not be conducted following state-of-the-art methods in clinical practice.

Alt-text: Unlabelled box dummy alt text

**What this paper adds**

• Knowledge and attitudes are currently most often examined or identified as important for pressure ulcer management in European hospitals.
• From an organizational perspective the availability of and access to clear standards and protocols was mentioned most frequently.
• Risk and skin assessment, repositioning, and the use of support surfaces are considered as important process aspects regarding pressure ulcer management in European hospitals.

Alt-text: Unlabelled box dummy alt text

## 1 Introduction

Pressure ulcer prevention and management belong to the core topics of nursing practice and research since decades (Griffiths and Norman, 2011; Kottner et al., 2024). Numerous systematic reviews, clinical practice guidelines, and good practice documents are available and are regularly updated to support evidence-based practice and to improve patient care and outcomes (Haesler et al., 2024; Patton et al., 2024; Serafin et al., 2025). Evidence further indicates that many of these guidelines are widely disseminated (El Genedy-Kalyoncu and Kottner, 2024).

However, pressure ulcer prevalence and incidence are still high in many care settings and age groups (Kottner et al., 2024; Li et al., 2020; Sugathapala et al., 2023; Triantafyllou et al., 2021), and projections indicate that pressure ulcer incidence is likely to increase globally in the future (Hu et al., 2025). This is particularly alarming because pressure ulcers can largely be regarded as preventable and are considered as a patient safety indicator. For example, the World Health Organization lists pressure ulceration as a common source of preventable patient harm (World Health Organization, 2025).

This indicates that pressure ulcer prevention and treatment may not be conducted following state-of-the-art methods in clinical practice. The question of why it is so difficult to implement and deliver evidence-based pressure ulcer practices is not new (Balzer and Kottner, 2015). For example, in the context of missed nursing care, Kalisch already pointed out in 2006 that repositioning is not performed as often as it should be (Kalisch, 2006) which is associated with higher pressure ulcer incidence (Alanazi et al., 2023). Results of recent systematic reviews identified numerous factors, facilitators, and barriers of pressure ulcer care in clinical practice (Heywood-Everett et al., 2023; Wan et al., 2023). However, due to the complexity of the various interacting and often setting-specific variables it is impossible to determine a single best way to improve care.

For hundreds of years, hospitals have been a critical part of health service delivery and are regarded as reservoirs of clinical resources and knowledge (World Health Organization, 2025). In the European Union there are approximately 2.3 million hospital beds which is on average 500 hospital beds per 100,000 people (eurostat, 2024). Due to the acuity and severity of diseases and invasive treatments, pressure ulcer prevention and treatment play a central role in hospital care, but evidence suggests that implementation is particularly challenging in this setting (Cordina et al., 2025; Li et al., 2020; Wan et al., 2023). As there is an unmanageable variety of strategies and studies on improving pressure ulcer practice, we wanted first to describe and map the available evidence regarding pressure ulcer prevention and treatment practices in European hospitals. The following specific review questions were developed: What pressure ulcer prevention or treatment interventions are provided/delivered at the bedside? What is the evidence regarding knowledge, skills, attitudes, opportunities, or motivation regarding pressure ulcer prevention and treatment of nurses, medical doctors and other health professions?

## 2 Methods

### 2.1 Protocol and registration

A scoping review protocol was developed and registered on 4 February 2025 (Linstedt et al., 2025).

### 2.2 Eligibility criteria

The Population, Concept, Context scheme (Peters et al., 2022) was used to structure the eligibility criteria: Population: healthcare professionals (incl. nurses, medical doctors); Concept: pressure ulcer prevention (incl. risk assessment) and treatment practice, knowledge, attitudes, skills, adherence to (quality) standards and guidelines, clinical reasoning and decision-making regarding pressure ulcer prevention and treatment; Context: hospitals in the European Union including all units and wards treating adult (18+ years) patients. Due to the close cooperation, comparable framework conditions, transnational laws, and common political objectives (European Union, 2025), the focus was placed on countries of the European Union. The United Kingdom, Norway, and Iceland were considered eligible due to the geographic proximity and historical reasons. Primary empirical data including qualitative and descriptive quantitative evidence, descriptive studies including cross-sectional and longitudinal designs, surveys, interviews, secondary data analyses published between 1 January 2014 and 31 December 2024 were included.

Exclusion criteria were: non-research articles including narrative reviews, letters, opinion papers, editorials; interventional research including trials, quasi-experimental studies, quality improvement projects, before-after-studies; healthcare settings other than hospitals; wards, settings treating pediatric patients < 18 years.

### 2.3 Information sources

The electronic databases Embase and Medline (via OVID) and CINAHL (via EBSCO Host) were searched. The reference lists of all potentially eligible articles were screened.

### 2.4 Search

The applied search strings including limits are shown in full detail in the published protocol (Linstedt et al., 2025) and in the Supplementary Material 1.

### 2.5 Selection of sources of evidence

Records identified from the databases were imported into the software EndNote 2025 (Clarivate, USA) and duplicates were removed. Screening and eligibility assessment were conducted by two reviewers independently using the software Rayyan (USA).

### 2.6 Data charting process

Data were extracted by one reviewer and cross checked by two additional reviewers. Iteratively developed data extraction tables were used for qualitative and quantitative evidence.

### 2.7 Data items

For qualitative studies data items included authors, year, country, setting, objectives, methods, sample and main results. The item ‘main results’ included overarching topics and themes identified in the original qualitative studies. For quantitative and mixed methods studies the additional item ‘main variables’ was applied.

### 2.8 Critical appraisal of individual sources of evidence

No risk of bias assessment was conducted.

### 2.9 Synthesis of results

Themes and concepts identified in the qualitative and mixed-methods studies and variables and associated quantitative results from the quantitative and mixed-methods studies were inductively and iteratively clustered into categories. If, for example, knowledge was identified as an important theme in qualitative studies and empirical results from knowledge tests were reported in quantitative studies, this was placed under the knowledge category in each case. The resulting categories and constructs were further inductively assigned to into the two overarching aspects structure and process (Donabedian, 1966). The structure aspect includes setting specific variables, including but not limited to administrative aspects, equipment, or qualification of staff. The process aspect includes activities and procedures regarding assessment, diagnostic decision-making, or the delivery of interventions (Donabedian, 1966).

Finally, all terms were inductively assigned to meaningful superordinate structural or process aspects. For example, concepts such as types of hospitals, types of wards, or the availability of guideline committees or experts were assigned to the category ‘system’ within ‘structure’. Repositioning, heel protection, or use of support surfaces was classified as ‘preventive interventions’ following the latest international clinical practice guidelines (Haesler et al., 2024). The identification of themes and the inductive assignment to categories were conducted independently by two reviewers.

## 3 Results

### 3.1 Selection of sources of evidence

**Fig. 1:**
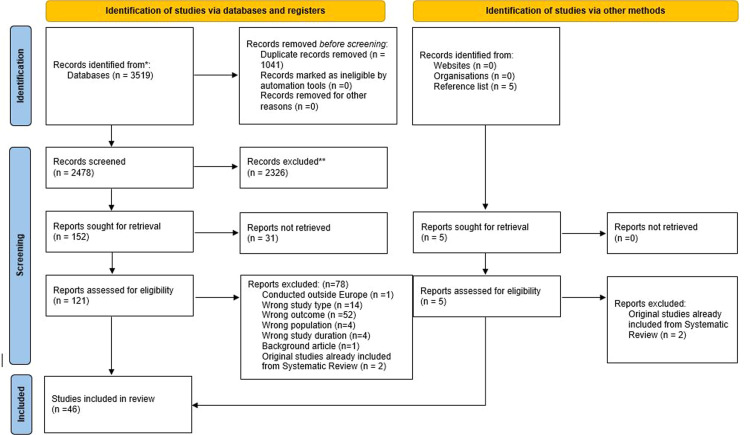
Study flow chart.

The selection of studies is shown in the flow diagram in Fig. 1. After exclusion of duplicates, 2478 articles were screened and 121 articles assessed in full text. In addition, 5 potentially eligible publications were assessed from the citation searching. Finally, 46 studies were included. Reasons for excluded articles are shown in the Supplementary Material 2.

### 3.2 Characteristics of sources of evidence

Included studies are described in detail in the Supplementary Material 3. Overall, studies were included from 18 countries. The majority were conducted in Sweden (*n* = 9), in the United Kingdom (*n* = 6), followed by Norway (*n* = 4), Spain (*n* = 4), and Germany (*n* = 4). Five reports included data from more than one country (Celik et al., 2024; Crunden et al., 2022; Johansen et al., 2023; Moore et al., 2015) including countries from outside the European Union (Celik et al., 2024; Crunden et al., 2022).

The study results reported either related to the entire hospital or to specific areas. The most frequently mentioned settings were internal medicine wards (*n* = 10), intensive care (*n* = 8), and surgical wards (*n* = 6). Areas such as oncology, operating rooms, or palliative care wards were only mentioned once (Table 1).

**Table 1:** Settings mentioned in included studies.

Setting	Number of times cited
Internal	10
Intensive care	8
Surgical	6
Orthopaedic	5
Geriatric	4
Neurology/Neurosurgery	3
Trauma	3
Rehabilitation	2
Emergency department	2
Paraplegic care	1
Palliative care	1
Oncology	1
Operating Room	1

There were 8 qualitative, 34 quantitative and 4 mixed-methods studies. All 8 qualitative studies used different interview techniques and included various health care professionals (see Supplementary Material 3, Table 1). The quantitative studies applied mainly cross-sectional designs including audits and surveys (see Supplementary Material 3, Table 2). Mixed-methods studies combined semi-structured interviews with survey or structured observational data (see Supplementary Material 3, Table 3).

**Table 2:** Structural aspects.

Aspects	Qualitative evidence (number of times identified)	Quantitative evidence (number of times investigated)	Mixed methods evidence (number of times mentioned)	Total
System level				
Health boards	-	1	-	1
Type of hospital (e.g. university versus general hospital, different NHS trusts)	-	2	-	2
Hospital size	1			1
Type of ward, department, specialty	-	4	-	4
Work shifts (morning, evening, night)	-	1	-	1
Organisation of prevention in institution	1	-	-	1
Existence of guidelines, expert committees	-	1	-	1
Availability of pressure ulcer experts	-	1	-	1
Electronic documentation systems, IT infrastructure	2	-		2
Availability/access to clear standards/protocols for prevention and treatment (incl. for different specialties)	4	2	1	7
Access to education, articles, participation in conferences, staff education	1	3	-	4
Patient safety culture (incl. quality score)	1	2	-	3
Staffing levels and staff shortage	3	1	-	4
Audits	-	1	-	1
Equipment				
Availability and adequacy of preventive equipment (e.g. support surfaces)	3	1	-	4
Costs of equipment	2	1	-	3
Efficient use of material (including oversupply)	1	1	-	2
Leadership				
Clear definition/allocation of responsibility	1	-	1	2
Team				
Time resources	2	1	-	3
Professional qualification, e.g. students, tissue viability nurses, advanced practice nurses	3	1	-	4
Availability of consultations of wound care specialists	1	-	-	1
Workload	1	-	-	1
Teamwork, incl. multidisciplinary team	2	2	1	5
Openness	1	-	-	1
Fear of negative consequences, e.g. reporting of too many pressure ulcers, blame culture, institutional repercussions	2	-	1	3
Individual level				
Awareness, attention	2	3	-	5
Knowledge (prevention, treatment, scores)	8	11	-	19
Attitudes (including scores)	2	9	-	11
Education, qualification (incl. postgraduation)	4	6	-	10
Interest, commitment, responsibility, priority, (incl. competing priorities)	4	2	1	7
Confidence in effectiveness of prevention	-	2	-	2
Work experience	-	2	1	3
Skills	-	1	-	1

**Table 3:** Process aspects.

Aspects	Qualitative evidence (number of times identified)	Quantitative evidence (number of times identified)	Mixed methods evidence (number of times mentioned)	Total
Roles				
Physician not involved in prevention but treatment	1	-	-	1
Prevention is part of role and responsibility of therapy staff	-	-	1	1
Communication/documentation				
Physicians and nurses use different language and documentation	1	-	-	1
Communication between junior and senior staff	1	-	-	1
Communication between theatre, wound nurses, therapists	1	1	1	3
Communication and documentation about prevention	2	2	-	4
Communication and documentation about treatment	2	2	-	4
Risk assessment				
Identification of patients at risk (including at admission, within certain time periods)	1	9	-	10
No reassessment of low-risk patients	1	-	-	1
Way of risk assessment strategy, e.g. different instruments, ‘clinical eye’, clinical judgement	1	5	1	7
Assessment of nutritional status	-	2	-	2
Skin assessment				
Conduct of skin assessment	-	10	-	10
Early identification of skin changes	-	2	-	2
Documentation of skin assessment	-	1	-	1
Preventive interventions				
Documented care plan, care plan in use	-	2	-	2
Patient information, counselling, education	-	3	-	3
Shared decision making	1	-	-	1
Some patients too sick to turn	1	-	-	1
Repositioning (importance, frequency)	1	8	-	9
Adherence to planned repositioning	-	5	-	5
Use of support surfaces	-	6	1	7
Support surfaces more often used when people know how to use it	1	-	-	1
Heel protection, off-loading	-	4	1	5
Individualised prevention	-	-	1	1
Nutritional interventions	-	2	-	2
Referral to dietician	-	1	-	1
Skin care and protection	-	5	-	5
Treatment interventions				
Pressure ulcer classification used incorrectly, incl. differences between nurses and physicians	1	1	-	2
Underreporting, lacking documentation	-	1	-	1
Use of prevention devices (e.g. heel protection) when PU already exists	1	-	-	1
Higher PU categories associated with support surface use, heel protection, planned repositioning	-	1	-	1
Nutrition	-	1	-	1

### 3.3 Critical appraisal within sources of evidence

A risk of bias assessment was not done.

### 3.4 Results of individual sources of evidence

#### 3.4.1 Qualitative evidence

Detailed results of the included qualitative studies are shown in Supplementary Material 3 (Table 1). Semi-structured interviews identified that, for example, awareness and personal interest (Gaspar et al., 2022; Hommel et al., 2017), positive attitudes (Crunden et al., 2022), knowledge and education (Crunden et al., 2022; Greenwood et al., 2023; Hommel et al., 2017) are important for adequate pressure ulcer prevention. After implementation of a pressure mapping system, Gunningberg et al. (2018) described, that physicians are not involved in pressure ulcer prevention but treatment only. Differences between professions also include the use of different language and documentation systems (Gaspar et al., 2022). Pressure ulcer risk assessment approaches were described in three studies (Acosta-Hernandez et al., 2023; Gaspar et al., 2022; Hultin et al., 2022). Themes including staffing, time, workload, and the availability of equipment were also identified (Crunden et al., 2022; Gaspar et al., 2022). Based on results of six focus groups Johansen et al. (2023) reported that pressure ulcer prevention and treatment have less priority in intensive care compared to therapies that ensure survival.

#### 3.4.2 Quantitative evidence

Detailed descriptions of the included quantitative studies are shown in Supplementary Material 3 (Table 2). Various variables ranging from structural aspects such as the availability of local standards to pressure ulcer prevention and treatment activities were described. For example, standardized knowledge tests were applied in nine studies and all studies unanimously conclude that pressure ulcer knowledge was inadequate (e.g. (Charalambous et al., 2019; de Almeida Tavares et al., 2015; De Meyer et al., 2019; Gunningberg et al., 2015). Nine studies used standardised attitude measurement instruments and results revealed moderate to high attitude scores towards pressure ulcer prevention (e.g. (Charalambous et al., 2019; Halasz et al., 2021; Lopez-Franco et al., 2020; Vivero et al., 2023).

Prevalence studies and surveys investigated the frequencies of applied preventive interventions. For example, numerous studies reported low proportions of patients receiving pressure ulcer risk and skin assessments, repositioning, or off-loading in patients at pressure ulcer risk (e.g., (Bredesen, 2015; Hoviattalab et al., 2014; Källman et al., 2022; Moore et al., 2015; Stephenson et al., 2021). Results from prevalence studies in Sweden also indicate associations between the category of existing pressure ulcers and the frequencies/proportions of applied preventive/therapeutic interventions: the higher the pressure ulcer category the more interventions were applied (Baath et al., 2014).

#### 3.4.3 Mixed-methods evidence

Results of the mixed-methods studies are described in Supplementary Material 3 in Table 3. Based on case studies Balzer et al. (2014) describe that pressure ulcer risk assessment using standardized instruments differs from clinical judgement. Using a realist evaluation approach Coleman et al. (2024) described how the use of structured risk assessment may help to overcome a blame culture in the hospital and that pressure ulcer prevention equipment is not always tailored to the individual needs. Worsley et al. (2017) described that physiotherapist and occupational therapists are not involved in pressure ulcer prevention and there is a lack of communication with nursing staff.

### 3.5 Synthesis of results

A total of 33 structural aspects were identified in all included studies, which were assigned to the categories system level, equipment, leadership, team, and individual level (Table 2). The categories of knowledge and attitudes were examined or identified by far the most frequently, at 19 and 11 times respectively. Formal education and qualification were mentioned 10 times and personal interest and/or commitment 7 times. At the system level, the availability of and access to clear standards or protocols was mentioned most frequently (*n* = 7), followed by access to educational opportunities including participation at conferences or reading articles (*n* = 4), and staffing (*n* = 4). Access to adequate material and teamwork were mentioned with the same frequency of 4 times each. A total of 11 aspects were identified only once, such as hospital size, institutional organization of prevention, or the availability of guidelines/expert committees.

The summary of process aspects is presented in Table 3. Thirty-two themes were identified and assigned to the categories: roles, communication/documentation, risk assessment, skin assessment, preventive interventions, and treatment interventions. The activities of risk assessment (*n* = 10) and the type of risk assessment (*n* = 7), skin assessment (*n* = 10), repositioning (*n* = 9) and the use of support surfaces (*n* = 7) were by far the most frequently covered topics followed by adherence to planned reposition (*n* = 5), heel off-loading (*n* = 5), and skin care (*n* = 5). Themes of interprofessional communication and documentation were mentioned several times, including between different professions or between junior and senior staff.

## 4 Discussion

### 4.1 Summary of evidence

The overall objective of this scoping review was to describe and synthesize available evidence about pressure ulcer prevention and treatment practices in European hospitals, in particular in hospitals in the European Union. More than 60 themes and aspects were identified in the included studies which were assigned to structure and process according to Donabedian (1966).

Review findings indicate that pressure ulcer management structure includes various organizational (e.g. type of documentation system), setting-related (e.g. type of ward), but also workforce-specific (e.g. workload) variables. However, the concepts of knowledge, attitudes, and education were mentioned most often in qualitative and quantitative research. This may indicate that these themes are considered of utmost importance regarding pressure ulcer practice. Indeed, knowledge and attitudes might be considered as important components of capability enabling a certain behavior (Michie et al., 2011) or clinical decision to provide or to omit care (Kalisch et al., 2009). A striking finding across all included studies was that the level of knowledge is insufficient (e.g. Bjurbo et al., 2024, Cukljek et al., 2022, Halasz et al., 2021). A critical lack of pressure ulcer knowledge and especially a difference between the level of perceived versus actual knowledge was observed also in other settings (Hayes et al., 2023) which is a strong indication of potential for improvement.

From an organizational perspective, the availability of standards or protocols was mentioned most often followed by access to education, availability of equipment, staffing levels, and teamwork mainly based on qualitative evidence. These factors, lying outside the individual healthcare professional, may also influence the opportunity that a certain behavior will be exhibited (Kalisch et al., 2009; Michie et al., 2011) although the strengths and directions of associations of these variables with the outcome pressure ulceration seem to be inconsistent (Alanazi et al., 2023).

The pressure ulcer management process most often investigated include risk assessment, skin assessment, repositioning, off-loading, and skin care. These aspects are in line with the current guideline-based approach to pressure ulcer prevention and treatment (Haesler et al., 2024) and evidence indicates that these interventions provided by nurses in hospitals are effective to prevent pressure ulceration (Alanazi et al., 2023; Mattila et al., 2025). However, interprofessional communication between different healthcare professions and documentation and teamwork also gained substantial attention in the included studies indicating that pressure ulcer care depends on an interprofessional approach (Hayes et al., 2023; Kalisch et al., 2009).

Factors identified in this scoping review including resources, knowledge, or teamwork are similar to general aspects to be considered during intervention implementation or quality improvement (McArthur et al., 2021). This supports the assumption that multifaceted strategies are more effective than single strategies to improve care (Cassidy et al., 2021; Hulscher and Wensing, 2020).

Review results further indicate, that the majority of research focuses on the entire hospital or larger settings such as internal and surgical wards and intensive care units. However, the emergency department or the operating room are also high-risk settings (Fulbrook et al., 2019; Lupe et al., 2013; Yao et al., 2025). Based on high number of included studies, it is very surprising that there is so little research on pressure ulcer management practice in these areas.

### 4.2 Limitations

Although we wanted to map the evidence of actually delivered pressure ulcer prevention or treatment interventions, we also included studies providing evidence about environmental and structural aspects, because the availability of certain organizational features is likely to influence clinical pressure ulcer practice. The data extraction and determination of categories followed an inductive approach without following a particular theory or model. This approach was chosen to capture the wide heterogeneity of reported aspects. However, this might limit the reproducibility of the identified themes and categories. Due to historical, political, and legislative reasons we focused on countries of the European Union only. However, we included also studies reporting results of countries outside the European Union (Celik et al., 2024; Crunden et al., 2022). It is unclear whether our findings are generalizable to other geographical regions. Because of the descriptive nature of a scoping review we did not conduct a risk of bias assessment. Although we describe empirical findings, we are unable to evaluate the internal or external validity, nor we support relationships between structure and process features.

### 4.3 Conclusions

More than 60 organizational, structural and process characteristics were identified potentially influencing pressure ulcer practice in European hospitals. Individual knowledge and attitudes, as well as the availability of standards/protocols, were most often investigated, indicating a perceived priority in shaping pressure ulcer practice. Formal qualifications, access to education, and availability of adequate equipment can be also regarded as important. Risk and skin assessment, repositioning, and the use of support surfaces have received the most attention from a process perspective and can be regarded as evidence-based strategies to prevent and treat pressure ulcers. Since all studies show knowledge deficits and a lack of implementation of basic prophylactic measures, there seems to be high potential to improve pressure ulcer prevention and to enhance patient safety. Moreover, the observed increase in prophylactic measures for existing pressure ulcers also indicates that prevention is being implemented too late. Future studies are therefore needed to investigate whether, for example, increased pressure ulcer knowledge actually leads to better care processes and outcomes.

The review results also suggest that pressure ulcer prevention and treatment can only take place in functioning interprofessional teams with good communication. Every project or trial trying to improve pressure ulcer care must therefore consider and involve all professional groups.

The themes and categories identified in our scoping review might be further used to design quality improvement projects or studies, and/or to evaluate pressure ulcer management in hospitals including designing audits. Review results also identified areas in which a large number of individual studies are available (e.g. knowledge, attitudes). Therefore, systematic reviews would be justified to examine the quality of the evidence more thoroughly.

## Funding

This work was partly supported financially by Baxter SAS (France). The funding had no influence on any aspect of the presented work.

## CRediT authorship contribution statement

**Jan Kottner:** Conceptualization, Data curation, Formal analysis, Funding acquisition, Investigation, Methodology, Project administration, Resources, Software, Supervision, Validation, Visualization, Writing – original draft, Writing – review & editing. **Ulrike Linstedt:** Data curation, Formal analysis, Investigation, Validation, Writing – review & editing. **Ahmed Tafesh:** Data curation, Formal analysis, Investigation, Validation, Writing – review & editing. **Monira El Genedy-Kalyoncu:** Data curation, Formal analysis, Investigation, Supervision, Validation, Writing – review & editing.

## Declaration of competing interest

This review has been partly supported financially by Baxter SAS (France). The funding had no influence on any aspect of the presented work. The first author of this manuscript is Associate Editor of the International Journal of Nursing Studies and member of the Guideline Governance Group of the International Guidelines for the Prevention and Treatment of Pressure Injuries.

## Appendix Supplementary materials

Image, application 1

Image, application 2

Image, application 3
