# Supplementary material for: Pressure ulcer practice in European hospitals: a scoping review
Source: Int J Nurs Stud Adv. 2025 Dec 23;10:100477. doi: 10.1016/j.ijnsa.2025.100477 (PMC12861148; doi:10.1016/j.ijnsa.2025.100477)
Supplement: Supplementary file 1 [file mmc1.docx]

**Supplementary Material 1**

**Article title:** Pressure ulcer practice in the European Union: a scoping review

**Authors:** Jan Kottner, Ulrike Linstedt, Ahmed Tafesh, Monira El Genedy-Kalyoncu

**Corresponding author:** Prof. Jan Kottner

jan.kottner@charite.de

Charité Universitätsmedizin Berlin,

Institute of Clinical Nursing Science,

Charitéplatz 1, 10117 Berlin, German

| **Embase and MEDLINE (OVID)** | | | |
| --- | --- | --- | --- |
|  | # | Searches |  |
| Population | 1 | (nurse or paramedical personnel or nursing staff or (physician or medical personnel) or health care professional or "health care professional" or (personnel or named groups by occupation) or ("nurs*" or "physician*" or "staff" or "assistant*" or "personnel")).ab,ti. | 2892264 |
| Concept pressure ulcer | 2 | ("pressure ulcer*" or "pressure sore*" or "pressure injur*" or "bedsore*" or "bed sore*" or "decubitus").ab,ti. | 50594 |
|  | 3 | 1 and 2 | 11376 |
| Concept practice | 4 | (attitude or knowledge or skill or nursing competence or staff training).ab,ti. | 2534603 |
|  | 5 | 3 and 4 | 1761 |
|  | 6 | (motivation or behavior or conation or engagement or extrinsic motivation or incentive or intrinsic motivation or motivational intensity or participation or education).ab,ti. | 4006222 |
|  | 7 | ("attitude*" or "knowledge" or "competence" or "training" or "motivation" or "behave*r").ab,ti. | 4146339 |
|  | 8 | (protocol or quality control or protocol compliance or health care quality or patient safety).ab,ti. | 1411333 |
|  | 9 | ("quality" or "compliance" or "experience*" or "perception*" or "awareness" or "attention" or "patient safety").ab,ti. | 9377211 |
|  | 10 | (clinical decision making or decision making or clinical decision rule or evidence based practice or evidence based medicine or evidence based nursing).ab,ti. | 587455 |
|  | 11 | (evaluation or program evaluation).ab,ti. | 3839105 |
|  | 12 | (clinical practice or professional practice or procedures).ab,ti. | 2310565 |
|  | 13 | ("practice" or "care" or "evaluation" or "implement*" or "service").ab,ti. | 11193606 |
|  | 14 | (disability assessment or risk or risk management or risk reduction or sentinel event or risk assessment or assessment).ab,ti. | 10015641 |
|  | 15 | ("risk" or "assessment*").ab,ti. | 10295995 |
|  | 16 | (prevention or prophylaxis or treatment or therapy).ab,ti. | 18054541 |
|  | 17 | 5 or 6 or 7 or 8 or 9 or 10 or 11 or 12 or 13 or 14 or 15 or 16 | 37768139 |
| Context | 18 | hospital or hospital management).ab,ti. | 3598527 |
|  | 19 | "hospital*".ab,ti. | 4660759 |
|  | 20 | emergency ward.ab,ti. | 2835 |
|  | 21 | Operating room or Operating theatre).ab,ti. | 95959 |
|  | 22 | ("emergency ward*" or "emergency room*" or "operating room*" or "operating theatre").ab,ti. | 178124 |
|  | 23 | 18 or 19 or 20 or 21 or 22 | 4767903 |
|  | 24 | 3 and 17 and 23 | 5207 |
| Limits | 25 | limit 24 to human | 4796 |
|  | 26 | limit 25 to humans | 4796 |
|  | 27 | limit 24 to yr="2014 - 2024" | 3149 |
| Total | 28 | remove duplicates from 27 | 1876 |

Search conducted on 21^st^ January 2025

**Database – CINAHL (EBSCOhost)**

AB ( nurse or nurses or nursing or physician or nursing staff or health care professional or registered nurse ) AND AB ( pressure injuries , or pressure ulcers , or pressure sores , or bedsores , or bed sores , or decubitus ) OR AB ( attitudes or knowledge or skills or perceptions or opinions or thoughts or feelings or beliefs ) OR AB ( motivation or motivate or motivating or engagement or participation or self-determination or self efficacy ) OR AB ( clinical decision making or clinical decision-making skills or clinical judgement ) OR AB ( quality control or quality assurance ) OR AB ( disability assessment or risk assessment or risk reduction ) OR AB ( hospital or acute setting or inpatient or ward or emergency department or operating room or operating theatre ) Limiters - Abstract verfügbar; Erscheinungsdatum: 20140101-20241231; Forschungsartikel; Menschen; Altersgruppen: All Adult; Location of Practice: Europe; Sprache: Dutch/Flemish, English, French, German, Italian, Norwegian, Portuguese, Spanish, Swedish Expanders - Entsprechende Themen anwenden Search modes - SmartText Searching Show Less

Limiters - Abstract Available; Publication Date: 20140101-20241231; Research Article; Human; Age Groups: All Adult; Special Interest: Advanced Nursing Practice, Patient Safety, Wound Care; Location of Practice: Europe; Language: Dutch/Flemish, English, French, German, Italian, Norwegian, Portuguese, Spanish, Swedish

Expanders - Apply equivalent subjects Search modes - Proximity

View Results (116)

Search conducted on 27th January 2025
