# Supplementary material for: Pressure ulcer practice in European hospitals: a scoping review
Source: Int J Nurs Stud Adv. 2025 Dec 23;10:100477. doi: 10.1016/j.ijnsa.2025.100477 (PMC12861148; doi:10.1016/j.ijnsa.2025.100477)
Supplement: Supplementary file 2 [file mmc2.docx]

**Supplementary Material 2**

**Article title:** Pressure ulcer practice in the European Union: a scoping review

**Authors:** Jan Kottner, Ulrike Linstedt, Ahmed Tafesh, Monira El Genedy-Kalyoncu

**Corresponding author:** Prof. Jan Kottner

jan.kottner@charite.de

Charité Universitätsmedizin Berlin,

Institute of Clinical Nursing Science,

Charitéplatz 1, 10117 Berlin, German

Reasons for exclusion

| **No.** | **Author, (year)** | **Title** | **Reason for exclusion** | **Number of excluded studies (total n=80)** |
| --- | --- | --- | --- | --- |
|  | Al- Ghraiybah et al. (2021) | The relationship between the nursing practice environment and five nursing-sensitive patient outcomes in acute care hospitals: A systematic review | Conducted outside Europe | 1/1 |
|  | Alanazi et al., (2022) | Systematic review: Nurses' safety attitudes and their impact on patient outcomes in acute-care hospitals | Wrong outcome | 1/52 |
|  | Alshahrani et al., (2021) | Nursing interventions for pressure injury prevention among critically ill patients: A systematic review | Background article | 1/1 |
|  | Bahlman-van Ooijen et al., (2024) | Low-value and high-value care recommendations in nursing: A systematic assessment of clinical practice guidelines | Wrong outcome | 2/52 |
|  | Barrois et al., (2017) | Prevalence, characteristics and risk factors of pressure ulcers in public and private hospitals care units and nursing homes in France | Wrong outcome | 3/52 |
|  | Belen and Duce, (2018) | Pressure ulcers, one more step in the care and safety of our patients | Wrong population | 1/4 |
|  | Borbala et al. (2023) | Health policy approach to the treatment and prevention of pressure ulcers | Wrong outcome | 4/52 |
|  | Borsting et al., (2018) | Prevalence of pressure ulcer and associated risk factors in middle- and older-aged medical inpatients in Norway | Wrong outcome | 5/52 |
|  | Brasaite et al., (2017) | Health Care Professionals' Knowledge Regarding Patient Safety | Wrong outcome | 6/52 |
|  | Chaboyer et al., (2024) | The effect of pressure injury prevention care bundles on pressure injuries in hospital patients: A complex intervention systematic review and meta-analysis | Wrong study design | 1/14 |
|  | Chamanga and Ward, (2015) | Documentation and record-keeping in pressure ulcer management | Wrong outcome | 7/52 |
|  | Deakin et al., (2023) | Medical and surgical nurses' approach to patient pressure injury prevention education: An integrative review | Wrong outcome | 8/52 |
|  | Downie et al., (2014) | Avoidable pressure ulcer rates in six acute UK Trusts | Wrong outcome | 9/52 |
|  | Dugaret et al., (2014) | Prevalence and incidence rates of pressure ulcers in an emergency department | Wrong outcome | 10/52 |
|  | Falk-Brynhildsen et al., (2022a) | Swedish translation, cultural adaptation and psychometric evaluation of the pressure ulcer knowledge assessment tool for use in the operating room | Wrong outcome | 11/52 |
|  | Falk-Brynhildsen et al., (2022b) | The Swedish version of the attitude towards pressure ulcer prevention instrument for use in an operating room context (APUP-OR): A nationwide psychometric evaluation | Wrong study design | 2/14 |
|  | Fiorini et al., (2016) | Wound Care Management: impact on outcomes of nursing care | Wrong outcome | 12/52 |
|  | Garcia et al., (2020) | Clinical results of the nurses of advanced practice in complex chronic wounds in Andalucia | Wrong outcome | 13/52 |
|  | Garcia et al., (2021) | Prevalence, incidence and risk factors for foot pressure ulcers in hospitalized elderly patients. An observational and prospective study | Wrong outcome | 14/52 |
|  | Gasperini et al., (2021) | Predictors of adverse outcomes using a multidimensional nursing assessment in an Italian community hospital | Wrong outcome | 15/52 |
|  | Georgieva, (2021) | Assessment of the risk of decubitus ulcers using Braden scale | Wrong outcome | 16/52 |
|  | Gillespie et al., (2014) | Repositioning for pressure ulcer prevention in adults | Wrong study duration | 1/4 |
|  | Giovannoni et al., (2024) | Complementing Braden scale for pressure ulcer risk with clinical and demographic-related factors in a large cohort of hospitalized Italian patients | Wrong study design | 3/14 |
|  | Gonzalez-Samartino et al., (2018) | Accuracy and completeness of records of adverse events through interface terminology | Wrong outcome | 17/52 |
|  | Granados-Plaza et al., (2021) | Association of Nursing Practice Environment on reported adverse events in private management hospitals: A cross-sectional study | Wrong outcome | 18/52 |
|  | Heikkila et al. (2022) | Validation of the Helsinki University Hospital prevent pressure Injury Risk Assessment Tool: a prospective observational study | Wrong outcome | 19/52 |
|  | Heywood et al., (2022) | The prevention of medical-device related pressure ulcers in a Critical Care Unit | Wrong outcome | 20/52 |
|  | Houmani et al., (2020) | Intensive Care Unit Risk Assessment: A Systematic Review | Wrong outcome | 21/52 |
|  | Hommel et al., (2020) | Exploring the incidence and nature of nursing-sensitive orthopaedic adverse events: A multicenter cohort study using Global Trigger Tool | Wrong outcome | 22/52 |
|  | Hultin et al., (2020) | PURPOSE T in Swedish hospital wards and nursing homes: A psychometric evaluation of a new pressure ulcer risk assessment instrument | Wrong outcome | 23/52 |
|  | Johansen et al., (2015) | Pressure Ulcer in Norway-A Snapshot of Pressure Ulcer Occurrence across Various Care Sites and Recommendations for Improved Preventive Care | Wrong outcome | 24/52 |
|  | Kambova et al., (2019) | The knowledge and practises of nurses in the prevention of medical devices related injuries in intensive care - questionnaire survey | Wrong outcome | 25/52 |
|  | Kennedy et al., (2018) | Ensuring healthy skin as part of wound prevention: an integrative review of health professionals' actions | Wrong study duration | 2/4 |
|  | Kroger et al., (2014) | Regional differences in the incidence of inpatients with pressure ulcers in Germany | Wrong outcome | 26/52 |
|  | Kurt et al., (2024) | Nursing interventions to prevent pressure injury among open heart surgery patients: A systematic review | Wrong study design | 4/14 |
|  | Lacey et al., (2019) | Preventative interventions, protocols or guidelines for trauma patients at risk of cervical collar-related pressure ulcers: A scoping review | Wrong study duration | 3/4 |
|  | Lawton et al., (2015) | Can staff and patient perspectives on hospital safety predict harm-free care? An analysis of staff and patient survey data and routinely collected outcomes | Wrong outcome | 27/52 |
|  | Lechner et al., (2017) | Dry skin and pressure ulcer risk: A multi-center cross-sectional prevalence study in German hospitals and nursing homes | Wrong outcome | 28/52 |
|  | Liu et al., (2023) | The knowledge and attitudes regarding pressure ulcer prevention among healthcare support workers in the UK: A cross-sectional study | Wrong outcome | 29/52 |
|  | Liukka et al., (2021) | Differences between professionals' views on patient safety culture in long-term and acute care? A cross-sectional study | Wrong outcome | 30/52 |
|  | Lopes et al., (2020) | Multicentre study of pressure ulcer point prevalence in a Portuguese region | Wrong population | 2/4 |
|  | Lopez L.R. et al., (2020) | Hospital prevalence of dependence-related lesions in the province of Burgos. Multicenter study | Wrong outcome | 31/52 |
|  | Lopez M. et al., (2022) | Auditing completion of nursing records as an outcome indicator for identifying patients at risk of developing pressure ulcers, falling, and social vulnerability: An observational study | Wrong outcome | 32/52 |
|  | Lospitao-Gomez et al., (2017) | Validity of the current risk assessment scale for pressure ulcers in intensive care (EVARUCI) and the Norton-MI scale in critically ill patients | Wrong study design | 5/14 |
|  | Lovegrove et al., (2021) | Comparison of pressure injury risk assessment outcomes using a structured assessment tool versus clinical judgement: A systematic review | Wrong study design | 6/14 |
|  | Lovegrove et al., (2018) | The relationship between pressure ulcer risk assessment and preventive interventions: a systematic review | Original Studies already included from Systematic Review | 1/4 |
|  | Lucchini et al., (2018) | Incidence and risk factors associated with the development of pressure ulcers in an Italian general intensive care unit | Wrong outcome | 33/52 |
|  | Ma et al., (2024) | Evaluation of the risk prediction model of pressure injuries in hospitalized patient: A systematic review and meta-analysis | Wrong outcome | 34/52 |
|  | Majda et al., (2024) | Perceptions of Clinical Adverse Event Reporting by Nurses and Midwives | Wrong outcome | 35/52 |
|  | Marchon and Maillard, (2023) | Epidemiology of pressure ulcers in Le Mans General Hospital between 1996 and 2019: Impact of a dedicated "Pressure ulcer, Wounds and Healing" task force | Wrong outcome | 36/52 |
|  | Meredith et al., (2024) | Nurse understaffing associated with adverse outcomes for surgical admissions | Wrong outcome | 37/52 |
|  | Monsonis-Fililla et al., (2021) | Improving risk assessment and prevention of pressure injuries during the implementation of a best practice clinical guideline | Wrong outcome | 38/52 |
|  | Moore et al., (2015) | Wound-care teams for preventing and treating pressure ulcers | Wrong study design | 7/14 |
|  | Munthlin Athlin et al., (2021) | Heel pressure ulcer, prevention and predictors during the care delivery chain - when and where to take action? A descriptive and explorative study | Wrong study design | 8/14 |
|  | Musa et al., (2021) | Clinical impact of a sub-epidermal moisture scanner: What is the real-world use? | Wrong study design | 9/14 |
|  | Nayar et al., (2021) | Waterlow score for risk assessment in surgical patients: a systematic review | Wrong outcome | 39/52 |
|  | Palese et al., (2019) | The activities/tasks performed by health care aids in hospital settings: a mixed-methods study | Wrong outcome | 40/52 |
|  | Pancorbo-Hidalgo et al., (2019) | Prevalence of pressure injuries and other dependence-related skin lesions in adult patients admitted to Spanish hospitals: The fifth national study in 2017 | Wrong outcome | 41/52 |
|  | Pancorbo-Hidalgo et al., (2014) | Pressure ulcers epidemiology in Spain in 2013: Results from the 4th National Prevalence Survey | Wrong outcome | 42/52 |
|  | Parvizi et al. (2024) | A systematic review of nurses' knowledge and related factors towards the prevention of medical device-related pressure ulcers | Original Studies already included from Systematic Review | 2/4 |
|  | Pinkney et al., (2014) | Why do patients develop severe pressure ulcers? A retrospective case study | Wrong outcome | 43/52 |
|  | Porcel-Galvez et al., (2022) | Predictive validity of the INTEGRARE scale in identifying the risk of hospital-acquired pressure ulcers in acute care hospital settings | Wrong outcome | 44/52 |
|  | Porter-Armstrong et al., (2018) | Education of healthcare professionals for preventing pressure ulcers | Wrong study design | 10/14 |
|  | Rapetti et al., (2023) | Pressure ulcers in hospital patients: incidence and risk factors | Wrong study design | 11/14 |
|  | Ravindra and Kathaliya, (2022) | Pressure Ulcer Prevention In Bedridden Patients: Evaluation Of Nursing Professional's Knowledge And Practice | Wrong population | 3/4 |
|  | Rasero et al., (2015) | Pressure Ulcers in Older Adults: A Prevalence Study | Wrong outcome | 45/52 |
|  | Reddy, (2015) | Pressure ulcers: treatment | Wrong outcome | 46/52 |
|  | Roberts et al. (2016) | Nurses' perceptions of a pressure ulcer prevention care bundle: a qualitative descriptive study | Wrong population/conducted outside Europe | 4/4 |
|  | Rodriguez-Nunes et al., (2019) | Nursing records, prevention measures and incidence of pressure ulcers in an Intensive Care Unit | Wrong outcome | 47/52 |
|  | Ryan et al., (2023) | Moisturizers, Emollients, or Barrier Preparations for the Prevention of Pressure Injury: A Systematic Review and Meta-Analysis | Wrong study duration | 4/4 |
|  | Sardo et al., (2015) | Pressure ulcer risk assessment: retrospective analysis of Braden Scale scores in Portuguese hospitalised adult patients | Wrong outcome | 48/52 |
|  | Sardo et al., (2024) | Pressure ulcers/injuries prevention in emergency services: A scoping review | Wrong outcome | 49/52 |
|  | Scafide et al., (2020) | Bedside Technologies to Enhance the Early Detection of Pressure Injuries: A Systematic Review | Wrong study design | 12/14 |
|  | Schneider and Geraedts, (2016) | Staffing and the incidence of pressure ulcers in German hospitals: A multicenter cross-sectional study | Wrong outcome | 50/52 |
|  | Skytt et al., (2016) | A longitudinal qualitative study of health care personnel's perceptions of simultaneous implementation of three risk assessment scales on falls, malnutrition and pressure ulcers | Wrong study design | 13/14 |
|  | Suarez et al., (2014) | Detection of adverse events in an acute geriatric hospital over a 6-year period using the global trigger tool | Wrong outcome | 51/52 |
|  | Sving et al., (2020) | A multifaceted intervention for evidence-based pressure ulcer prevention: a 3 year follow-up | Wrong study design | 14/14 |
|  | Wiseman et al., (2024) | The impact of whole of patient nursing assessment frameworks on hospital inpatients: A scoping literature review | Wrong outcome | 52/52 |
|  | Wan et al., (2023) | Barriers and facilitators to implementing pressure injury prevention and management guidelines in acute care: A mixed-methods systematic review | Original Studies already included from Systematic Review | 3/4 |
|  | Wu et al., (2023) | Barriers and facilitators to pressure injury prevention in hospitals: A mixed methods systematic review | Original Studies already included from Systematic Review | 4/4 |
