# Supplementary material for: Pressure ulcer practice in European hospitals: a scoping review
Source: Int J Nurs Stud Adv. 2025 Dec 23;10:100477. doi: 10.1016/j.ijnsa.2025.100477 (PMC12861148; doi:10.1016/j.ijnsa.2025.100477)
Supplement: Supplementary file 3 [file mmc3.docx]

**Supplementary Material 3**

**Article title:** Pressure ulcer practice in the European Union: a scoping review

**Authors:** Jan Kottner, Ulrike Linstedt, Ahmed Tafesh, Monira El Genedy-Kalyoncu

**Corresponding author:** Prof. Jan Kottner

jan.kottner@charite.de

Charité Universitätsmedizin Berlin,

Institute of Clinical Nursing Science,

Charitéplatz 1, 10117 Berlin, German

[Table 1 Qualitative Studies 2](#_Toc210916057)

[Table 2 Quantitative studies 7](#_Toc210916058)

[Table 3 Mixed Methods 27](#_Toc210916059)

Table 1 Qualitative Studies

| **No.** | **Author (Year)** | **Country** | **Setting** | **Objectives** | **Methods** | **Sample** | **Main results** |
| --- | --- | --- | --- | --- | --- | --- | --- |
| 1. | Hommel et al. (2016) | - Sweden | - Hospitals (n=6) | - Exploring successful factors to prevent PUs in hospital settings | - Interviews, semi-structured (n=13) - Focus groups, semi-structured (n=6, 4-8 persons/group) | - Managers, physicians, registered nurses (n=39) | - Creating a good organization   - Easier for small hospitals to develop and sustain an effective organization of prevention work   - Good IT systems for documentation, quality registers are helpful - Maintaining persistent awareness   - Personnel with knowledge of the concept of quality improvement in health care   - Action plans established on every ward - Realizing the benefits for patients, increased patient safety   - Personnel have evidence-based knowledge to prevent PUs, include scientific and practical knowledge   - Education on pressure-relieving mattresses and aids, support surfaces are more often in use when personnel knows how to use   - Person-centred, allowing the patients to choose, patient cooperation is important for success |
| 2. | Gunningberg et al. (2018) | - Sweden | - University hospital - Geriatric/internal Medicine | - Describing staff’s perceptions of a continuous pressure mapping system to prevent PU in a hospital ward | - Focus groups, semi-structured (n=7) | - Participants (n=19) - RN (n=6) - Assistant nurses (AN) (n=6) - Physical therapists (n=3) - Physicians (n=2) - Assistant nurse managers (n=2) | - Physicians not involved in PU prevention, trust in RNs and ANs - Physicians are called, when PU occurred and orders for treatment were needed - Awareness of the need for PU prevention increased among staff - Easier to make patients understand and participate in PU prevention because of monitoring system |
| 3. | Gaspar et al. (2021) | - Portugal | - Hospital | - Understanding perceptions of nurses regarding PU prevention practice and examine what is needed to improve patient safety | - Focus group, semi-structured - Content analysis | - Nurses (n=11) - Female (n=6) - Clinical nurse specialist (n=8) - Nurse manager (n=4) - Professor/researcher (n=3) - Advanced education in tissue viability (n=7) | - Lack of PU risk assessment   - Missed nursing care because of understaffing   - Lack of knowledge about health policies   - No reassessment of low risk patients during hospitalization   - Low value on documentation, no interest   - No responsibility, lack of leadership - Different records of doctors and nurses   - Different clinical language   - Classification system is not used correctly - Prevention and treatment   - No adequate equipment e.g. support surfaces and beds   - High level on personal interest and involvement needed - PU prevention means team approach |
| 4. | Hultin et al. (2021) | - Sweden | - University hospital - Nursing homes (n=2) | - Evaluating the clinical usability of PURPOSE-T among registered nurses | - Focus groups, semi-structured (n=6) - Psychometric evaluation | - Nurses (n=29) - Female (n=27) - Age range 23 to 65 years - Nursing experience: 6 months to 34 years - 5 nurses with master degrees in nursing | - Compared to Modified Norton Scale, more benefits, skin assessment included - Easy to learn and understand - Better awareness of risk factors - Time efficient - PURPOSE T increased nurses’ awareness of risk factors (e.g., sensory perception, moisture, circulation, diabetes, medical devices). - Nurses stressed the need for PURPOSE T to be integrated into electronic health records for practical use. - Team collaboration with assistant nurses during assessments was reported as valuable. - Overall, PURPOSE T perceived as a potential replacement for older tools; further feasibility testing recommended. |
| 5. | Crunden et al.  (2022) | - UK (n=4) - Czech Republic (n=1) - USA (n=3) - Italy (n=2) - Hong Kong (n=1) - Thailand (n=1) - Switzerland (n=1) - Finland (n=1) - Australia (n=1) - Belgium (n=1) - Brazil (n=1) | - Hospitals - Pediatrics and Adults | - Identifying determinants of the practice of medical device-related pressure ulcers reporting using the Tailored Implementation for Chronic Disease checklist | - Interviews, semi-structured - Online via Skype and Zoom; - Via telephone, - Face-to-face - 30-60 minutes - Content analysis | - Representation of experts in tissue viability and wound assessment or reporting e.g. plastic surgeon, RN, Clinical nurse specialist in wound care, professor for nursing/head of quality of care | - Individual health professional factors   - Perception of negative consequences; worries about missing PU or reporting too many of these wounds = judged negatively   - Lack of knowledge relating to medical device-related PU   - Attitudes: focus on prevention of traditional PU; on ICU medical device-related PU are expected= no documentation - Professional interactions   - Openness and teamwork; PU as team problem, possibility to ask questions, receiving positive feedback, sharing good practice   - Difficulty for junior staff getting accepted from senior staff by reporting medical device-related PU   - Open communication between wound nurses and theatre nurses helps to understand positioning   - Learning from mistakes - Incentives and resources   - Device procurement: cost-driven, but feedback can have impact what devices are purchased by organisation   - Transparency in presenting PU can lead to negative organisation perceptions and financial repercussions, fear of litigation - Capacity for organisational change   - Documentation in electronic systems means high workload   - Lack of time: documentation only of serious issues   - Staffing: not every institution employs nurses specialized in tissue viability; struggle in educating staff - Main facilitators are education, openness and teamwork for reporting PU - Barriers: perception of negative consequences, lack of knowledge, negative attitudes, peer influence, financial disincentives, workload, lack of time and staffing |
| 6. | Acosta-Hernàndez et al. (2023) | - Spain | - University hospitals (n=2) - ICU | - Exploring attitudes, knowledge and perceived barriers by intensive care nurses regarding PU treatment and prevention in critical care setting | - Interviews, semi-structured - Interview guide 10 questions - Content analysis | - Nurses (n= 22) - Female n=14) - Age: mean 42 (SD 10.6) years - Work experience: mean 9 (SD 5.8) years - Education on PU e.g. postgraduate courses years ago: mean 4 (SD 3.9) years | - Lack of specific knowledge about PU in ICU   - No initial training at starting at ICU ward   - APN in ulcers in chronic wounds helpful, but others do not update themselves - Common risk assessment tools for PU are known, but not always recorded or used; more trust in “clinical eye” - Staff shortage: barrier in PU prevention in ICUs - Lack of consensus among professionals, no unification of criteria and treatment, missing information - Lack of communication in prevention of PU, only information about treatment - Skin care as important as vital sign, but depends on given priority - Areas of improvement   - Efficient use of material resources   - Easy access to continuous education courses or protocols regarding PU care   - Lack of well-trained personnel   - Effective transmission of verbal and written information |
| 7. | Johanson et al.  (2023) | - Norway - Iceland | - University hospitals (n=3) - ICU wards (n=6, 2 within each hospital) | - Exploring Intensive care nurses’ knowledge, experience and bedside practice in prevention and care of PU | - Focus groups (n=6) | - Intensive care nurses and registered nurses (n=25) - Age: 28 to 57 years - Nursing experience: 3 to 33 years | - ABCD (Airway, Breathing, Circulation, Disability assessment) have priority in ICU patients, but less priority in PU treatment - Experienced in moisture associated skin damage and fungal skin infections, but poor knowledge in PU treatment - Consultation of wound care specialist is needed, but not everywhere accessible - Skin focus: priority appear and disappear with projects - High-tech beds: not every ward has access to appropriate beds - All at risk of PU, some are too sick to turn, e.g. haemodynamic unstable - Nurses have different access to appropriate beds and mattresses - Skin is last on priority list in ICU after ABCD - The skin is higher on priority in patients in long-term than in short-term stays on ICU - Wound care or PU prevention did not occur in intensive care nursing education, but in basic nursing program: lack of focus on tissue injuries and wound care in postgraduate ICU education may explain lack of knowledge in PU among nurses |
| 8. | Greenwood et al. (2023) | - UK | - Hospitals | - Exploring how offloading devices are implemented and used in clinical practice for prevention of heel PUs in hospitals | - Interviews via telephone - 13 initial theories were refined into 3 program theories about how offloading devices are used “proactively” by nurses | - Tissue Viability Nurse Specialists (n=8) - Experience: 2.5 to 20 years | - Proactive use of devices for the prevention of heel PUs   - Specialist knowledge   - Accessibility of devices, pillows are used as an alternative device   - Repositioning, most important component - Reactive use of devices for the treatment of heel PUs and prevent further deterioration   - Using heel protection when PU already exists - Staff knowledge, identification of patients at risk, stock of devices and costs are influential mechanisms on device use |

1. Hommel, A., Gunningberg, L., Idvall, E. and Bååth, C. (2017), Successful factors to prevent pressure ulcers – an interview study. J Clin Nurs, 26: 182-189. <https://doi.org/10.1111/jocn.13465>
2. Gunningberg, L., Bååth, C., & Sving, E. (2018). Staff’s perceptions of a pressure mapping system to prevent pressure injuries in a hospital ward: A qualitative study. Journal of Nursing Management, 26(2), 140–147. <https://doi.org/10.1111/jonm.12526>
3. Gaspar S, Botelho Guedes F, Budri A, Ferreira C, Gaspar de Matos M. (2022). Hospital-acquired pressure ulcers prevention: What is needed for patient safety? The perceptions of nurse stakeholders. Scand J Caring Sci.; 36: 978–987. <https://doi.org/10.1111/scs.12995>
4. Hultin, L., Gunningberg, L., Coleman, S., & Karlsson, A.-C. (2021). Pressure ulcer risk assessment—registered nurses´ experiences of using PURPOSE T: A focus group study. J Clin Nurs; 31: 231–239. <https://doi.org/10.1111/jocn.15901>
5. Crunden, E., Worsley, P., Coleman, S. and Schoonhoven, L. (2022). Barriers and facilitators to reporting medical device-related pressure ulcers: A qualitative exploration of international practice, International Journal of Nursing Studies; 135, <https://doi.org/10.1016/j.ijnurstu.2022.104326>
6. Acosta-Hernández,C., Fernández-Castillo, R.-F., Montes-Vázquez, M. and González-Caro, M.-D. (2023). Is caring for pressure ulcers in the intensive care unit in Spain still a challenge? A qualitative study on nurses’ perceptions, Journal of Tissue Viability, 32, (1), 114-119, <https://doi.org/10.1016/j.jtv.2022.12.002>
7. Johansen E, Bredesen IM, Jónasdóttir RJ, Lind R. (2023). ABCD before E-verything else—Intensive care nurses' knowledge and experience of pressure injury and moisture-associated skin damage. Int Wound J. 20(2): 285-295. doi:10.1111/iwj.13872
8. Greenwood, C., Nixon, J., Nelson, E., McGinnis,E. and Randell, R. (2023). Offloading devices for the prevention of heel pressure ulcers: A realist evaluation, International Journal of Nursing Studies, 141, <https://doi.org/10.1016/j.ijnurstu.2023.104479>

Table 2 Quantitative studies

| **No.** | **Author**  **(Year)** | **Country** | **Setting** | **Objectives** | **Methods** | **Sample** | **Main variables** | **Main Results** |
| --- | --- | --- | --- | --- | --- | --- | --- | --- |
|  | Sving et al. (2014) | - Sweden | - Hospitals (n=2) - University hospital (1100 beds) - General hospital (350 beds) | - Investigating associations between variables on different levels in setting (hospital, unit, patient) and documentation of risk assessment, skin assessment within 24h of admission, use of pressure-reducing mattresses and planned repositioning in bed | - Prevalence study | - Patients (n=825) - University hospital (n=610, 73.9%) - Age, mean 68.4 (SD 16.8) years - Braden score <17 (n=143, 17.6%) | - Documentation of risk assessment - Skin assessment within 24h of admission - Pressure-reducing mattresses - Planned repositioning in bed - Age - Gender - Days of hospitalization - Braden risk score <17 - Hospital type - Unit type - Nurse staffing, total hours of care per patient day - Workload | - Prevalence of hospital-acquired PU - Planned repositioning was less likely to be used in university hospital 42% (university hospital) vs. 51.6% (general hospital) - Patients at university hospital have higher odds of having risk and skin assessment documented and of having pressure-reducing mattresses |
|  | Bååth et al.  (2014) | - Sweden | - Hospitals | - Prevalence and preventive interventions and pressure-reducing interventions | - Prevalence study - 3/2011 | - Patients (n= 16,466) | - Prevalence of pressure ulcers (overall, by categories) - Distribution of PUs categories (e.g., category I, II, III, IV) - Location of pressure ulcers - Stage of PUs at diagnosis - Measures for pressure relief (e.g., special mattresses, repositioning intervals) - Frequency and type of staff training - Implementation of prevention guidelines - Factors influencing prevalence (e.g., quality of care, staff awareness) | - Patients at risk get preventive interventions:   - Pressure-reducing mattress (76 to 81%)   - Heel protection (30 to 32%)   - Planned repositioning in bed (47 to 50%) - All patients with PU IV always had planned repositioning in bed - 1/3 of the patients received individual-planned repositioning in bed - Association between interventions and patients with PUs  \|  \| Category I (n= 3164) \| Category IV (n = 678) \| \| --- \| --- \| --- \| \| Mattress \| 66 to 73 % \| 85 to 89% \| \| Heel protection \| 24 to 29% \| 42 to 51% \| \| Planned repositioning \| 29 to 35% \| 43 to 59% \| |
|  |  |  |  |  | - 10/2011 | - Patients (n= 8265) |  |  |
|  |  |  |  |  | - 3/2012 | - Patients (n= 14,540) |  |  |
|  | Hoviattalab et al. (2014) | - Germany | - Hospitals (n=2) - Surgical - Medical | - Determining nurses’ PU prevention practice and the level of adherence to the recommendations of German Expert Standard for PU prevention (DNQP 2004) | - Observational descriptive - Survey | - Patients (n=32, 16 in each hospital, 8 in medical, 8 in surgical ward) on high PU risk, Braden score <12 - Female, n= 17, 53.1% - Surgical ward, n=17, 53.1% - Presence of PU, n=10, 31.2% | - Demographics, diagnosis, length of stay, PU history, and Braden scores. - Checklist of 27 preventive interventions, five themes - Risk assessment (2) - Skin inspection and care (9) - Positioning, mobilization and support surfaces (10) - Nutrition (3) - Education (3) - Each category: ‘observed’, ‘not observed’, ‘not applicable’ | - Preventive interventions of German National Expert Standard not fully implemented - Risk assessment on admission was undertaken in 34.3% (n=11) - Skin assessment less frequent, 15.6% - 28 (87.5%) of the patients had no record of skin inspection - Most frequent intervention were cleaning the skin (n=30, 93.8%) and minimum exposure to moisture (n=31, 98.8%), and skin protection during transfers (90.6%) - Less frequent interventions: Repositioning in less than half of the patients (40.6%), turning schedules (31.2%), heel off-loading (50%). Pressure-reducing mattresses used in less than half (43.8%) of patients. - Assessment and recording of nutritional status were observed very infrequently - One patient was informed about PU prevention - Patients at high risk do not receive adequate preventive PU care. - No significant differences in practices across morning, evening, and night shifts (p = 0.1). |
|  | De Almeida Tavares et al.  (2015) | - Portugal | - Hospitals (n=5) - University hospitals (n=2)   > 1000 beds   - Hospital centers (n=3), 300 – 600 beds | - Exploring knowledge and attitudes of nurses about four common geriatric syndromes: PU, incontinence, restraint use and sleep disturbance | - Survey - Online and   paper pencil | - Nurses (n= 1068) - RN in direct care position, serving older adults - Gender: Female 79.7% - Age: mean 34.1 (SD 8.5) years - Work experience: mean 11.3 (SD 8.4) years | - Geriatric Nursing Knowledge/Attitudes (GNKA), subscale of Geriatric Institutional Assessment Profile (GIAP) - Measurement of knowledge and attitudes of RN: - PU prevention, incontinence care, restraint use and management of sleep disturbance - 32 statements, 5-point Likert scales - 3 items: staff knowledge, hospital’s education support and work with older adults as burdensome, range 0 to 1 | - Knowledge score: mean 0.41 (SD 0.15) - 21.2 % (226/1068) knowledge score > 0.5 - PU management: mean 0.47 (SD 0.26) - Attitude score:   Mean 0.4 (SD 0.21)   - Positive attitude for management of PU: mean 0.57 (SD 0.37) - Nurses working in academic hospitals demonstrated significantly higher knowledge level than nurses in hospital centers: mean 0.50 (SD 0.18) vs. 0.48 (SD 0.20) |
|  | Gunningberg et al. (2015) | - Sweden | - Hospitals (n=3) - Wards:   surgical, orthopedic,  medical   - Palliative | - Describing and comparing knowledge of RN, AN and SN about preventing PUs | - Survey | - RN (n=196) - AN (n=97) - SN (n=122) | - Pressure ulcer knowledge assessment tool. (PUKAT), 26 MC questions, 6 themes: aetiology and development (5), classification and observation (5), risk assessment (2), nutrition (1), reduction of the magnitude of the pressure and shearing (7), reduction of the duration of pressure and shearing (5) - Range 0 (=no knowledge) to 26 (= excellent knowledge) - Mean knowledge score > 60% considered satisfactory - 5 MC questions added about behavior in clinical practice | - Prevention knowledge for all three groups was unacceptable or borderline (RN: 59.3%, AN: 55.4%, SN:61.0%) - Highest knowledge for “Nutrition” (83.1%) and “Risk Assessment” (75.7%) - Lowest knowledge for “Reduction in the amount of pressure and shear” (47.5%) and “Classification and observation” (55.5%) |
|  | Bredesen et al., (2015a) | - Norway | - Hospitals (n=6) - Somatic wards (n=88) | - Investigating the prevalence of PUs, patient-related risk factors, the use of preventive measures and how much the PU variance is at patient, ward and hospital level | - Prevalence study - Teams of two trained nurses collected data | - Patients (n=1209) - Female: 44.1% (n=533) - Age > 70 years, 40% (n=473) | - Braden score, range 6 (= high risk) to 23 (= no risk), <17 PU risk - Skin observation for PU location and category - PU preventive measures including the type of pressure-reducing support surfaces, frequency of repositioning in bed and chair | - HAPU prevalence 15% (n=182) - Difference for patients >70 years, more PUs, higher risk at getting PUs - 25.2% (n=305) at risk, Braden score <17, 51.1% (n=156) received no pressure-reducing mattresses and no planned repositioning, 17.7% (n=54) received both - Half of the patients with PU were identified as being at risk by Braden scale - Implications for practice: hospitals and ward management must facilitate improvements in level of PU attention and knowledge among staff in relation to preventive measures including the use of support surfaces and repositioning |
|  | Bredesen et al. (2015b) | - Norway | - Hospitals (n=4) - Wards (n=84) - ICU - Surgery, - Internal medicine, - Rehabilitation | - Comparing ward levels in PU prevalence and preventive measures | - Survey | - Patients (n=1056) - Age: >70 years, 38.2 % - Female: 45.3 % | - Hospital acquired pressure ulcer - PU prevention implementation, 3 subcategories: repositioning (no planned, every 2,3 and 4 hours), support surfaces (standard mattress, non-powered or powered redistributing mattress), elevated heels (yes/no) | - PU Prevention implemented, 75% on ICU wards - Association between patient safety culture and PU prevalence |
|  |  |  |  |  |  |  | - Safety Attitude Questionnaire (SAQ), 36 items covering 6 dimensions: teamwork (6), safety climate (7), perceptions of management (10), job satisfaction (5), stress recognition (4), working conditions (4), 5-point Likert scale, range 0 (= most negative) to100 (= most positive) | - Patient safety culture scores differ between wards, lowest on rehabilitation ward (52.7) and highest on surgery/inner medical ward (81.3), on ICU highest mean score (71.6 (3.6)) |
|  | Moore et al. (2015) | - Norway - Ireland | - Hospitals (n=2) | - Determining the difference in PU prevalence and prevention practices in two clinical settings, using formal structured risk assessment and other using clinical judgement only | - Survey - Norway: clinical judgement - Ireland: structured risk assessment tool | - Patients total (n=180) | - Risk assessment on admission - Skin protection for incontinent patients - Pressure redistribution devices - Documented repositioning care plan - Education of PU prevention practices | - Use of risk assessment tools draws attention to the need for PU risk assessment - Higher prevalence of PU in Norway: lack of available equipment and lack of attention for PU prevention  \| Prevention practice \| Norway (n=59) \| Ireland (n=121) \| \| --- \| --- \| --- \| \| Risk assessed on admission \| 8% (n=5/59) \| 85% (n=103/121) \| \| Documented nutritional screening \| 29% (n=10/34) \| 62% (n=28/45) \| \| Documented referral to dietician as required \| 5% (n=1/20) \| 47% (n=28/59) \| \| Documented PU  prevention care plan \| 24% (n=8/34) \| 62% (n=28/45) \| \| Skin protection for  Incontinent patients \| 13% (n=1/8) \| 39% (n=15/38) \| \| Pressure redistribution device in use (dynamic mattresses) \| 0% (n=0/59) \| 22% (n=27/121) \| \| Pressure redistribution device in use (chair) \| 7% (n=4/59) \| 9% (n=11/121) \| \| Documented repositioning care plan (bed) \| 15% (n=5/34) \| 56% (n=25/45) \| \| Documented repositioning care plan (chair) \| 0% (n=0/34) \| 13% (n=6/45) \| \| Education for the patient on PU prevention practices \| 7% (n=4/59) \| 0% (n=0/121) \| |
|  | Van Dishoeck et al. (2016) | - Netherlands | - Hospital | - Exploring the relation between the occurrence of PU or incontinence dermatitis and the quality of preventive care | - Matched case-control study - Data from a prior cross-sectional study on PU prevalence measurement in April 2010 (n = 453 patients) - Audit panel of 15 experts, 5 teams of 3 experts to evaluate the patients reports | - Patients (n=132, 44 cases, 88 controls) - Male, 61% - Age mean, 60 years - PU category I-II, 66% | - Eight guideline- based criteria + criterion of ‘non-recommended intervention.’: - Risk assessment, using risk assessment tool - Provision of patient information of PU risk and prevention - Turning and repositioning - Use of support surfaces to prevent heel PUs - Use of alternating-pressure active support replacement mattresses - Skin protection from exposure to excessive moisture - Adequate nutrition and - Skin assessment - Quality score for suboptimal factors for PU, 0 to 3. - Patient variables: age, gender, type of illness, comorbidities, ADL dependency, ICU stay, number of care problems, PU risk score | - Quality score (p = 0.032) and the PU risk score (p = 0.018) were associated with the occurrence of PU/IAD - Differences in risk assessment, prevention of heel PUs and use of alternating-pressure relieving mattresses. - Even with optimal care, some PU/IAD cases still occurred, showing patient risk factors also play a strong role. |
|  | Clark et al.  (2017) | - UK, Wales | - Hospitals (n=66) - NHS Trusts (n=3) - Health Boards (n=7) | - Identifying prevalence of PU and IAD - Exploring the appropriateness of pressure   redistributing mattresses to patients with, or at risk of PU | - Audit - Skin assessment by teams of two independent experienced nurses | - Patients (n=8365) - Female: 4659 (55.7%) - Medium or high risk: 4282 (51.7%) | - Pressure Sore Prediction Score (PSPS) - Prevalence of PUs - Severity of PUs according to categories I to IV - Location of PUs - Risk Factors for PUs (e.g. mobility, nutrition, moisture) - Patient demographics - Preventive Measures (e.g. mattresses, repositioning) | - Health Boards use different risk assessment tools - Differences between the Health Boards mainly stem from how effectively they implement prevention measures and train their staff - Boards with better training, use of specialized equipment, and consistent prevention strategies tend to have lower l proportions of PUs - Those with less focus on these areas show higher prevalence. - Proper staff education and proactive prevention are key to reducing PUs across the boards |
|  | Gonzàlez-Méndez et al. (2017) | - Spain | - Hospital - ICU | - Determining the incidence and risk factors of PU in critical care patients | - Prospective cohort-study - Daily direct observation by trained nurses plus patient record review. | - Patients (n=335) - Age, mean 59.8 (SD 14.3) years - Male, n =206, 61.5% | - Risk factors developing a PU - Length of stay - Prognostic variables: - the Braden score for PU risk and Simplified Acute Physiology Score III (SAPS). - Usage of support surfaces - Duration of invasive/non-invasive ventilation - Outcome: Development of PU (stages I–IV), location, time to onset. | - Preventive protocol has influence on low incidence of PU, repositioning every 2 hours - Multidisciplinary team including physicians, nurses and physiotherapists is involved in preventive - Although incidence was lower than many international studies, severity, complications, and immobilization patterns were strong predictors.  \| **Incidence** \| **Severity** \| **Location** \| **Time of onset** \| **Risk factors** \| \| --- \| --- \| --- \| --- \| --- \| \| 8.1% (27 patients developed PUs); \| 40.6% Stage I \| Sacrum most frequent (59.4%) \| Median = 7 days (range 1–34) \| Higher SAPS III score (HR = 1.04, p = 0.010) \| \| incidence rate = 11.72 PUs per 1,000 ICU patient-days \| 59.4% Stage II \| followed by heel (12.5%) \| 88.9% occurred within first 14 days \| In-hospital complications (HR = 6.48, p = 0.002) \| \| none Stage III/IV \|  \| Longer immobilization was paradoxically protective (HR = 0.42, p < 0.001) \| |
|  | Eglseer et al. (2019) | - Austria | - Hospitals (n=30) - Wards (n=202) | - Assess the fulfillment of structural indicators, the application of nursing interventions as process indicators and the prevalence as outcome indicators to PUs | - Survey - Structural indicators assessed by hospital nurse managers and ward head nurses. - Process and outcome indicators assessed bedside by two trained nurses via patient interviews and medical record review. | - Patients (n=2878) - Female, 51.4% (n=1480) - Age, mean 65.2 (SD 17.6) years | - Nursing Quality Measurement 2.0 - Skin inspection - Structural indicators - Process and outcome indicators | \| Process in PU prevention or treatment \| Organizational level, hospitals (n=30) \| Ward level (n=202) \| \| --- \| --- \| --- \| \| Interventions for PU prevention and treatment were conducted frequently \| Guidelines and multidisciplinary expert committees for PU care: 80% (n=24) of the hospitals \| Refresher course  for caregivers,  14.6 % (n=30) \| \| Patients with PU (n=95), 81.1% of these participants had moisturizer/barrier cream \| Regular audits  ensure  compliance with  protocol/  guideline, 59.4% (n=151) \| \| Repositioning was in 51.1% of the participant frequent. \| Risk patients  discussion  within a  multidisciplinary team  74.7 % (n=151) \| |
|  | De Meyer. et al. (2019) | - Belgium | •Hospitals (n=16)   - University hospital (n=1) - General hospitals with university character (n=4) - General hospitals (n= 11)   • Wards (n=29: ICU n= 8, geriatrics n= 13, rehabilitation n = 8) | - Measuring the knowledge of nurses and nursing assistants about pressure ulcer prevention | - Survey | - Nurses (n=430) and nursing assistants (n=43) - Female n=403 (88.0%) - Education: secondary school (n=39(8.5%), higher professional education (n=124 (27.1%), Bachelor degree (n=277 (60.5%), Master degree (n=14 (9.1%) | - Pre-final version of Pressure Ulcer Knowledge Assessment Tool 2.0 (PUKAT 2.0),28 MC questions, 6 themes, range 0 (= no knowledge, high deficit) to 28(= max. knowledge) - Total knowledge score of 17 (60 %) or higher is needed for satisfactory level | - Total score: mean 50.7% - Lowest scores: knowledge about prevention (42.7%, SD 17.5%), etiology (45.6%, SD 17.2%), prevention for specific patient groups (46.6%, SD 24.4%); - Highest scores at “risk assessment” (83.8%) and “nutrition” (59.6%, SD 29.5 %) - differences between wards: lower scores on rehabilitation wards than on ICU and geriatrics - Higher education level associated with higher knowledge scores except for “risk assessment” and “specific patient groups” |
|  | Biz et al. (2019) | - Italy | - Hospital - Orthopedic and Trauma Units (n=3) | - Identifying impact of nursing care in PU prevention in treatment of femoral fractures - Evaluating the knowledge related to the use of traction for hip fractures and their preoperative nursing management | - Survey | - Orthopedic nursing staff (n=70) - Orthopedic and trauma consultant (n= 40) - Orthopedic and trauma residents (n=26) - Age: mean 40 years - Male (n=78, 57.4%) - University degree (85.3%) | - Use of traction, skin traction or skeletal traction for stabilizing the femoral fracture - Pain management, - hygiene care, - VTE prevention, - Pressure ulcer prevention - 12-item questionnaire | - Preoperative traction for hip fractures is still in use, skin traction as well as skeletal traction - Traction negatively impacts pain control, hygiene, venous thromboembolism, and pressure ulcer prevention. - Consequences for PU prevention: Skin traction is better rated than skeletal traction, but new evidence shows, that traction in general is not recommended anymore, no positive impact on patient outcome - Traction makes PU prevention more difficult for nurses - Younger surgeons tend to use less traction; older surgeons often adhere to traditional practices |
|  | Hödl et al. (2019) | - Austria | - Hospitals (n=56) | - Identifying the degree of agreement between the Braden scale and clinical view regarding PU risk in mobile/chairfast/bedfast patients and subsequent preventive measures | - Prevalence study | - Patients (n=5274) - Age: mobile (n=4467), 63.9; chairfast (n=446), 74; bedfast (n=361), 74 years - Female, 53% | - Nursing quality measurement 2015 - PU risk assessment via Braden scale - Preventive measures: - Skin barrier cream - Information about PU - Relief of the heels - Repositioning in bed or chair - Prevention/treatment of dehydration and malnutrition - Visco-elastic Mattress - Cold foam mattress - Foam pillow | \|  \| Mobile \| Chairfast \| Bedfast \| \| --- \| --- \| --- \| --- \| \| Braden scale \| 20,9 % (n=934) \| 99.6 % (n=444) \| 100 % (n=361) \| \| Clinical judjgement \| 5 % (n=223) \| 61.7% (n=275) \| 86.1 (n=311) \|  - 45.6% of all the patients, who were at risk via Braden scale are also assessed for risk via clinical judgement - 20% of the patients got preventive measures although there were not at risk in both risk assessment ways - 50% of the patients, who are at risk via Braden scale, are not assessed at risk via clinical judgement, so implication for practice is using a validated risk assessment instrument in combination with clinical judgement for chairfast and mobile patients |
|  | Charalambous et al. (2019) | - Greece, Cyprus | - Hospital - ICU - Orthopedic - Paraplegic - Medical - Neurosurgery | - Evaluating knowledge and attitudes of nurses towards pressure ulcer prevention | - Survey - Paper pencil | - Nurses (n= 102), - Female, (n= 63, 61.8%) - RN (n=95, 93.1%) - ICU (n=45, 44.1%) - Familiar with national guidelines regarding PU prevention and treatment (n=69, 67.6%) | - Pieper Pressure Ulcer Knowledge Test (PUKT) - 44 questions with three answer, in this study, adapted version with 21 questions, options (yes, no, I don’t know), range not reported - 90% correct answers mean adequate knowledge | - Mean 16.16 (SD 2.55) - 77% correct answers (inadequate knowledge) - Knowledge and attitude correlated positively, better attitude levels mean better knowledge levels, R^2^=0.054 (Pearson's R=0,223, p=0,019), |
|  |  |  |  |  |  |  | - Attitudes questionnaire by Moore, 2004: 11 5-pointLikert scale questions, range 11 (= poor attitude) to 55 (=good attitude) | - Median 41.82 (SD 6.26) overall positive attitude - Nurses in ICU have better attitude (median 44, IQR 42 to 46) in comparison to other nurses, (median 42, IQR 39 to 45) p=.013´ - Nurses had inadequate knowledge but positive attitudes; educational programs and frequent reassessment recommended to improve PU prevention. |
|  | Lichterfeld-Kottner et al. (2020) | - Germany | - Nursing Homes - Hospitals | - Analyzing sex-specific differences in PU prevention and treatment of institutional-acquired PUs | - Prevalence study | - Patients in hospitals (n=58760, 60.3%) - Female (n=31631, 53.8%) - Age: Mean:66.7 (SD 18.0) | - Demographics and general information - Mobility - Care dependency with Care Dependency Scale - Pressure Ulcers - PU prevention (done or not done) | - PU prevention measures, differences between sexes:  \|  \| Women (n=31,631) \| Men (n=25,741) \| \| --- \| --- \| --- \| \| Soft positioning \| 3845 (12.2%) \| 2566 (10.0%)  p <0.001 \| \| Sheep skin \| 185 (0.6%) \| 115 (0.4%)  p<0.001 \| \| Regular skin assessment \| 10478 (33.1%) \| 7422 (28.8%)  P<0.001 \| \| Heel protection \| 1165 (3.7%) \| 783 (3.0%)  P <0.001 \| \| Counselling of patients \| 7082 (22.4%) \| 4985 (19.4%)  P <0.001 \| |
|  | Lopez-Franco et al. (2020) | - Spain | - Hospitals (n=7) | - Exploring the association of the attitude of PU prevention, with other factors | - Psychometric evaluation | - Nurses (n=438) - Female (n=354, 80.8%) - Registered Nurses (n=266, 60.7%) - Assistant nurses (n=161, 36.8%) - Work experience - Specific training in PU prevention, none (n=67, 15.3%), basic (n=93, 21.2%), multiple (n=278, 63.5%) | - Attitudes towards Pressure Ulcer Prevention tool APuP - 13 items, 5 categories, scores range from 13 (= poor attitude) to 52 (= good attitude), - > 39 (75%) means a positive attitude - Spanish version 12 items, range from 12 (= poor attitude) to 48 (= good attitude), | - Correlation (R=0.32) between knowledge and attitude - Higher attitude scores in RNs (mean 40.55, SD 3.98) compared to ANs (mean 39.08, SD 4.45), p= 0.001 - Higher attitude score in nurses with more training in PU prevention (mean 40.68, SD 3.92) compared to those, who received only basic training (mean 38.63, SD 4.88) |
|  | Gaspar et al. (2020) | - Portugal | - Hospital - Medical - Surgical - Orthopedics | - Describing and analyzing PUs problem based on data from hospital electronic health records | - Retrospective cohort-study | - Patients (n=3904) - Age, >65 years, 66% (n=2575) - Female, 57.5% (n=2244) - Hospital admission way through emergency department, 54.2% (n=2117) | - Visual Skin assessment - Risk assessment with Braden Scale - Admission way, e.g. Emergency Department | - Categorization and localization identification of PUs are different between nurses and doctors - Healthcare professionals need to have more awareness to skin inspection, that - when performed daily provide a major opportunity to improve PUs risk assessment and early identification of changes in skin status |
|  | Strube-Lahmann et al. (2021) | - Germany | - Hospitals - Emergency Department | - Revealing the current PU prevention-related processes and structures | - Survey | - Hospitals (n=276) - 201-500 beds, 40% - <200 beds, 38% - >1000 beds, (n=5, 1.8%) | - Questionnaire with 5-point-Likert scale about structures and processes according PU management - Questions about: - Specific personnel in response for PU prevention (medicine, nursing, other) - Internal guidelines for risk assessment and implementation of PU prevention - Documentation of measures with high degree of evidence | - Staff responsible for PU prevention, at least one PU manager n=175, 63.4%; (25 to medical sector, 172 to nursing sector, 13 to others); n=96, 34.8% had no PU manager - Prevention process, ‘skin inspection’ was performed very often (77.5% of n=262); ‘mobilization in bed’ (64.8% of n=265) and ‘30° tilt positioning’ (64.8% of n=250) - 30.8% of the hospitals had a guideline for recording PU risk in Emergency Department, more than half (55.8%) did not have such a guideline, 13.4% did not respond to this question - Risk assessment with PU guideline “yes” mean 4.4 (SD 0.8) vs. without PU guideline ‘no’ mean 3.1 (SD 1.5) |
|  | Eglseer et al. (2021) | - Austria | - Hospitals (n=91) - Nursing Homes (n=49) - Other institutions (n=9) | - Measuring improvement of quality of pressure ulcer care | - Prevalence study, 10 year-period | - Patients | - Austrian version of LPZ questionnaire | \|  \| 2009 (n=1724) \| 2018 (n=3382) \| \| --- \| --- \| --- \| \| Prevalence \| 4.4% \| 2.9% (p=.006) \| \| Moisturizing cream \| 77.4% \| 80.7% (p=not reported) \| \| No intervention for patients at risk \| 13.9% \| 2.6% (p<.01) \| \| Repositioning \| 42.9% \| 42.1% (p=not reported) \| \| Floating heels \| 41.8% \| 56.3% (p<.001) \| \| Client education \| 35.3% \| 44.1% (p<.01) \| \| Prevention/  treatment of nutritional deficiencies \| 28.7% \| 41.% (p<.001) \| |
|  | Gress Halasz et al. (2021) | - Slovakia | - Hospitals - Surgical - Medical - ICU - Geriatrics - Trauma - Neurology - Physiotherapy - Oncology | - Exploring knowledge and attitudes of nurses towards the prevention of PU | - Survey | - Nurses (n=225) | - Pressure Ulcer Knowledge Assessment Tool PUKAT, - 26 MC questions, 6 themes   Mean knowledge score >60% considered satisfactory   - Range = 0 (= no knowledge, high deficit) to 26 (= max. knowledge) | - Mean knowledge score 11.84 (SD 2.83) 45.5% - 9% (n=21) reached >60 % - Best knowledge score was nutrition (77.0%) - Least knowledge was risk assessment (38.5%) - Positive correlation between knowledge and attitudes - Higher education associated with higher knowledge |
|  |  |  |  |  |  |  | - Attitudes towards Pressure Ulcer Prevention tool APuP - 13 items, 5 categories, scores range from 13 (= poor attitude) to 52 (= good attitude), > 39 (75%) means a positive attitude | - Attitudes mean score 67.9% - 56.4% (n=127) reached satisfactory level >75 % |
|  | Källman et al. (2021) | - Sweden | - Hospitals | - Describing PU prevalence and prevention interventions over a 10-year period | - Prevalence study | - Patients (n=14,257 (2011); (n=11,358 (2020) | - Used preventive interventions - Risk assessment with Modified Norton Scale - Skin assessment | \|  \| 2011 \| 2020 \| \| --- \| --- \| --- \| \| PU prevalence \| 17.0% \| 11.4% \| \| Patients at risk getting preventive interventions \| 74.4% \| 96.2% \| \| Risk assessment in patients within 24hours: \| 14.1% \| 51.1% \| \| Skin assessment in patients within 24 hours: \| 32.4% \| 70.2% \| |
|  | Parisod et al. (2021) | - Finland | - Primary and specialized care | - Evaluating nursing staff knowledge about evidence-based PU prevention practice | - Survey | - Nurses (n=554) - Registered nurses. Bachelor’s degree (n=272, 49.45%) - Practical nurses, vocational level (n=238, 43.27%) - Primary Care (n=329, 61%) | - Pressure Ulcer Prevention Knowledge test (PUPK):   terms in 7 domains, PU development and risk factors (1), PU classification (2), PU risk assessment and PU prevention (3) with repositioning (4), pressure relief devices (5), skin assessment and skin care (6) and nutrition (7), each 5 items with ‘yes’, ‘no’, I don’t know, range 0 (= no knowledge) to 35 (= excellent knowledge) | - PUPK test mean 24.4, SD 4.09 - One person reached maximum score of 35 - Highest knowledge on PU risk assessment, (mean 4.42, SD 0.84) - Lowest knowledge in PU classification (mean 2.92, SD 0.96), PU prevention with repositioning (mean 2.87, SD 0.80) and PU prevention with pressure relief devices (mean 2.76, SD 1.05) - Knowledge differences regarding education (p=0.0139), higher education mean better knowledge in PU prevention - Differences in PU prevention knowledge based on work experience (p=0.0103), >14 years of work experience have higher knowledge level than those with <6 years of work experience - Self-evaluation of PU prevention skills correlate with knowledge levels |
|  |  |  |  |  |  |  | - Attitude towards pressure ulcer prevention (APuP) - 13 items, 5 categories, scores from 13 (=poor attitude) to 52 (= good attitude), > 39 (75%) means a positive attitude |  |
|  | Haavisto et al. (2021) | - Finland | - Hospitals - Primary Care - Specialised care | - Describing the use of consistent and evidence-based practices   in pressure ulcer prevention and to assess the validity and reliability of  the pressure ulcer prevention practice (PUPreP) instrument |  |  | - Pressure ulcer prevention practice (PUPreP) instrument, 42 items: risk assessment (9), skin assessment and skin care (9), nutrition (6), repositioning (9), pressure relief devices (4), documentation (5) | - Most used practice in PU prevention is repositioning - Skin assessment and skin care second most frequently used PU prevention practice - Least used prevention practice is nutrition - Attitudes towards PU prevention among participants correlate with PU prevention practices - Knowledge about PU associated with skin assessment and skin care practice (r=0.116) |
|  |  |  |  |  |  |  | - Pressure Ulcer Prevention Knowledge (PUPK), 0 (= no knowledge) to 35 (= excellent knowledge) - Attitude towards pressure ulcer prevention (APuP) - 13 items, 5 categories, scores from 13 (= poor attitude) to 52 (= good attitude), > 39 (75%) means a positive attitude |  |
|  | Stephensen et al. (2021) | - UK, England | - Hospitals (n=36) - NHS Trusts (n=18) | - Assessing the prevalence of PUs and measuring the adherence to elements of the aSSKINg framework and NICE PU standards | - Audit - Skin assessment by two independent trained nurses | - Patients (n=10,144) - 51.5% female | - aSSKINg framework - NICE Pressure Ulcer standards - Risk assessment tools documented (Waterlow, Braden, PURPOSE T, PURAT, others). - Outcome: PU prevalence (≥1 PU per patient). - PU characteristics: Category, location, device-related vs non-device-related. - Risk assessments: Tool used, time to completion, presence of care plan, repositioning regimen | - Risk assessment within 6 hours in 69.9% (n=7086) of the patients - Skin assessment by audit team, 88.1% (n=8490) - Care plan in use for patients at risk, 81%, (n=6576)  \| **PU Prevalence** \| 9.04% overall (917 patients with ≥1 PU) \| \| \| \| \| Range across trusts: 3.9% to 27.7% \| \| \| \| \| --- \| --- \| --- \| --- \| --- \| --- \| --- \| --- \| --- \| --- \| \| **Categories** \| 1: 18.2% \| 2: 45.8% \| 3: 11.1% \| \| 4: 4.6% \| \| Deep Tissue Injury: 10.9% \| \| Unstageable: 9.4% \| \| **Most frequent Location** \| Sacrum (29.5%) \| \| \| Buttocks (30.4%) \| \| \| \| Heels (13.2%) \| \|  - Planned repositioning in 51.4% (n=5216), but it varies between the NHS Trusts between 18 and 100% among those, 40.7% had 2-hourly, 44.1% had 4-hourly repositioning schedules. - Different risk assessment instruments in use: Braden (21.3%), Glamorgan, Maternity, PURAT, PURPOSE-T (9.44%), Waterlow (56.6%); standardizing the approach for PU risk assessment would be beneficial - Equipment in use, tendencies for over-equipment are observed. - PURPOSE T had shortest average completion time (4.65 hours) compared to Waterlow (7.84) and Braden (7.20). - Patient Education: Only 43.6% received PU prevention information (37.0% verbal, 6.6% written). - Equipment Use: Over-prescription noted: higher-spec mattresses given more often than needed by risk score. PU incidence 2–3× higher in patients with equipment than those without - Patient Education: Only 43.6% received PU prevention information (37.0% verbal, 6.6% written). - Overall conclusion: The aSSKINg bundle was inconsistently applied, particularly in **repositioning** and **patient education**. |
|  | Avgerinou et al. (2022) | - Greece | - Hospital - ICU (n=3) | - Investigating knowledge of ICU nurses for prevention and treatment of PUs | - Survey | - Nurses (n=107) - Female (n=86, 80.4%) - Postgraduate studies (MSc., n=48, 44.9%) | - Sparta Tool PU 2014, 3 subscales: knowledge (31), practices (11) and attitudes (13), 55 questions - PU knowledge: range 0 (no knowledge) to 31 (excellent knowledge) | - Postgraduate education is associated with higher knowledge score - Postgraduate education is associated with higher rating in knowledge subscale staging - Nurses are more familiar with treatment of PUs than in prevention |
|  | Cukljek. et al.  (2022) | - Croatia | - Hospital - Medical - Surgical - Neurological - Pediatrics | - Determining the knowledge of nursing students and nurses about pressure injury prevention | - Survey | - Nurses (n=114) and nursing students (n=198) (n=312 in total) - Female (n=252, 80.8%) - Surgical ward (n= 57, 50.0%) | • Pressure Ulcer Knowledge Assessment Tool (PUKAT 2.0): 28 MC questions (0= no knowledge (high deficit) to 28 = max. knowledge)   - Total knowledge score of 17 (60 %) or higher is needed for satisfactory level | - 15 (4.8%) respondents reached 60% of correct answers - Differences between two groups in total number of correct answers: clinical hospital nurses 45.48%, students 39.7% - Both groups unsatisfactory level of PU knowledge - Lowest knowledge in both groups in prevention of PUs - Highest level in risk assessment |
|  | Andersson et al. (2022) | - Sweden | - Hospital - Internal Medicine | - Describing and analyzing the documentation of PUs | - Review of medical records | - Patients (n=1460) - Female, n=798 (54.7%) | - Risk assessment - Label - Localization - Categorization of PU | - Point prevalence measurement:14 patients - All patients lack documented risk assessment - Skin assessment within 24hr was performed in 10/14 patients (71.4%) - Routine of registration of PUs within 24hr, 2/3 of the patients lacked documentation within the medical records - Uniform documentation within medical records facilitates high-quality healthcare delivery |
|  | Eirinidou et al.  (2023) | - Greece | - Hospital - Pathology - Surgery | - Evaluating nurses’ knowledge about prevention and treatment of PUs | - Survey | - Nurses (n=111) - Female (n=105, 94.6%) - Age: mean 39.2 (SD 8.1) years - Surgery (n=57, 51.4%) | - Questionnaire by Gouda et al. (2014): 27 questions about PU care, 0 (=no knowledge) to, 27 (= max. knowledge) | - Association between higher education and knowledge in five questions about treatment and prevention of PUs - Correct answers associated with departments: nurses from pathology ward had more correct answers than from surgery ward - Nurses have adequate knowledge about treatment and prevention of PUs. |
|  | Vivero et al. (2023) | - Spain | - Hospital - Traumatology | - Exploring attitudes and perceived barriers of nurses towards PU prevention | - Survey | - Health Care Professionals (n= 35 (21 nurses, 14 assistants) - Female: 94.3%, - Work experience < 5 years (71.4%). 34.3% <10 years - Technicians in Auxiliary Nursing Care (TCAEs) (n=14), Mean age 31–50 years (57.2%), - RNs are responsible for clinical assessment and planning of care and TCAEs provide basic nursing care and support under RN supervision | - Attitudes towards Pressure Ulcer Prevention Instrument (APuP):12 items, structured in 5 dimensions: 1) Priority of prevention, 2) Personal competence, 3) Impact of PU, 4) Confidence in prevention effectiveness, 5) Responsibility. Range from 12 (= poor attitude) to 58 (= good attitude). Higher scores indicate more positive attitudes | - Mean APuP score: 41.82 (SD 6.26), indicating generally positive attitudes, 82.46% of the professionals feel competent and capable to apply preventive measures, 94.25% acknowledge responsibility in PU prevention, 79.9% viewed prevention as a priority, 84.7% acknowledged high social and economic impact of Pus, and 87.1% felt confident PUs are avoidable with proper measures · |
|  |  |  |  |  |  |  | - Barriers to Pressure Ulcer Prevention (BPUPP): - 25 items, structured in 4 factors: 1) Equipment and organizational aspects, 2) Motivation and resources, 3) Knowledge, 4) Staff and collaboration. - Range 0 to 75 - Items rated as 'frequently' or 'always' are considered barriers | - 17 barriers identified, including lack of staff, time constraints, material costs, incomplete documentation, insufficient communication, differences between nurses and TCAEs: nurses identified 16 barriers, TCAEs identified 18 barriers, 14 were common to both groups. |
|  | Tervo-Heikkinen et al. (2023) | - Finland | - Hospitals (n=16) | - Exploring prevalence and preventive interventions of PUs | - Prevalence study | - Patients (n=5902) | - Skin status assessment (yes/no) - Support surface in use (yes/no) - Preventive skin care - Repositioning | \| PU prevalence \| 13% (n=747) in all PU stages \| \| --- \| --- \| \| PU risk assessment <8h after admission \| 30 % (n=1778) \| \| Skin status assessment at admission \| more frequently conducted in patients with high PU risk and with limited mobility \| \| Nutrition care \| more frequently implemented for patients with PU \| |
|  | Bjurbo et al.  **(**2024) | - Sweden | - Hospital - Geriatrics - Emergency Care - Internal Medicine | - Exploring knowledge of and attitudes towards PU prevention | - Survey | - Assistant nurses, (n= 88, female n= 74) - Department of geriatrics (n=42) - Department of Emergency Care and Internal Medicine (n=46) | • Pressure Ulcer Knowledge Assessment Tool (PUKAT 2.0),  28 MC questions (0= no knowledge (high deficit) tp 28 = excellent knowledge)   - Total knowledge score of 17 (60 %) or higher is needed for satisfactory level | - Correlation between PU prevention knowledge and PU attitude - 3 of all participants had a satisfactory level on knowledge |
|  |  |  |  |  |  |  | • Attitude towards Pressure Ulcer Prevention (APUP)   - 13 items, 5 categories, scores range from 13 (=poor attitude) to 52 (= good attitude), > 39 (75%) means a positive attitude | \| Personal competency to prevent PU \| lowest scores (mean 65.3 %, SD 17.9) \| \| --- \| --- \| \| Responsibility in PU prevention \| Highest scores (mean 87.0, SD 20.9%) \| \| Confidence in the effectiveness of prevention \| (mean 87.0, SD 21.5 %) \| \| Emergency Department and Internal Medicine (78.3, SD 13.6) \| Geriatrics (77.4, SD 19.5) \|  - Priority of PU prevention - Impact of pressure injuries - 58 participants (74.3%) equal or higher score than 75 % - Attitudes higher among those who had > 5 years work experience |
|  | Celik et al.  (2024) | - Croatia - Italy - Turkey | - Hospitals - Operating room | - Examining factors of operating room nurses’ attitudes and awareness towards surgery-related PU prevention | - Survey - Online | - Operating room nurses, at least undergraduate degree (n=258): - Italy (n=72, 37.9%) 62.5% female, state hospitals (81.9%), 66.7% had not received education on PU prevention - Croatia (n=66, 25.6%) female (89.4%) state hospitals (89.4%), 81.8% had not received education on PU prevention | - Nursing information form: 16 questions | - Association between awareness and attitudes of   Operating room nurses in Croatia (r = 0.453,)  Association between awareness and attitudes in Italy (r = 0.191)   - Difference between mean awareness scores of nurses by country according to the following descriptive characteristics: considering themselves sufficient, willingness to receive education, and reading articles in Croatia; having received education, willingness to receive education, participation in symposiums/conferences/congresses, and reading articles in Italy - **Awareness scores:** - Croatia: Mean 10.01 ± 3.24 (range 1-14) - Italy: Mean 9.51 ± 3.58 (range 1-14) - **Attitude scores:** - Croatia: Mean 37.48 ± 3.44 (range 29–44) - Italy: Mean 36.20 ± 4.02 (range 22–44) - Both below the threshold (≥39) for a positive attitude - **Barriers identified:** - Lack of education (Croatia 81.8%, Italy 66.7%) - Not using risk scales (Croatia 81.8%, Italy 87.5%) - Limited participation in scientific events (Croatia 75.8%, Italy 73.6%) - Low frequency of reading PI-related articles (Croatia 60.6%, Italy 41.7%) - **Factors associated with higher awareness/attitudes:** - Considering oneself sufficient (both countries, p < 0.05) - Reading articles (Croatia p = 0.040; Italy p = 0.006) - Participation in conferences (Italy p = 0.012) - Implementing preventive interventions (Croatia p = 0.023; Italy p < 0.001) |
|  |  |  |  |  |  |  | - Surgery-related PU Awareness Form: - 14 yes-no-questions to evaluate risk for surgery-related PUs: range 0-14 |  |
|  |  |  |  |  |  |  | - Attitudes towards Pressure Ulcer Prevention Instrument (APuP): - 13 items, 5 categories, scores range from 13 (poor attitude) to 52 (good attitude), > 39 (75%) means a positive attitude |  |

1. Sving, E., Idvall, E., Hogberg, H., & Gunningberg, L. (2014). Factors contributing to evidence-based pressure ulcer prevention. A cross-sectional study [Article]. International journal of nursing studies, 51(5), 717-725. <https://doi.org/https://dx.doi.org/10.1016/j>
2. Baath, C., Idvall, E., Gunningberg, L., & Hommel, A. (2014). Pressure-reducing interventions among persons with pressure ulcers: Results from the first three national pressure ulcer prevalence surveys in Sweden [Review]. Journal of Evaluation in Clinical Practice, 20(1), 58-65. <https://doi.org/https://dx.doi.org/10.1111/jep.12079>
3. Hoviattalab, K., Hashemizadeh, H., D'Cruz, G., Halfens, R. J., & Dassen, T. (2014). Nursing practice in the prevention of pressure ulcers: an observational study of German Hospitals [Article]. Journal of clinical nursing, 24(11-12), 1513-1524. <https://doi.org/https://dx.doi.org/10.1111/jocn.12723>
4. De Almeida Tavares, J. P., da Silva, A. L., Sa-Couto, P., Boltz, M., & Capezuti, E. (2015). Portuguese nurses' knowledge of and attitudes toward hospitalized older adults [Article]. Scandinavian journal of caring sciences, 29(1), 51-61. <https://doi.org/https://dx.doi.org/10.1111/scs.12124>
5. Gunningberg, L., Martensson, G., Mamhidir, A. G., Florin, J., Muntlin Athlin, A., & Baath, C. (2015). Pressure ulcer knowledge of registered nurses, assistant nurses and student nurses: A descriptive, comparative multicentre study in Sweden [Article]. International Wound Journal, 12(4), 462-468. <https://doi.org/https://dx.doi.org/10.1111/iwj.12138>
6. Bredesen, IM., Bjøro, K., Gunningberg, L., et al. (2015a). Patient and organisational variables associated with pressure ulcer prevalence in hospital settings: A multilevel analysis. BMJ Open, 5(7), e007584. <https://doi.org/10.1136/bmjopen-2015-007584>
7. Bredesen, I. M., Bjøro, K., Gunningberg, L., & Hofoss, D. (2015b). The prevalence, prevention and multilevel variance of pressure ulcers in Norwegian hospitals: a cross-sectional study. International journal of nursing studies, 52(1), 149–156. <https://doi.org/10.1016/j.ijnurstu.2014.07.005>
8. Moore, Z., Johansen, E., van Etten, M., Strapp, H., Solbakken, T., Smith, B. E., & Faulstich, J. (2015). Pressure ulcer prevalence and prevention practices: a cross-sectional comparative survey in Norway and Ireland. Journal of Wound Care, 24(8), 333-339. <https://doi.org/10.12968/jowc.2015.24.8.333>
9. Van Dishoeck A., Looman C.W.N., Steyerberg E.W., Halfens R.J. G. & Mackenbach J. P. (2 0 1 6) Performance indicators; the association between the quality of preventive care and the prevalence of hospital-acquired skin lesions in adult hospital patients. Journal of Advanced Nursing 72(11), 2818–2830. doi: 10.1111/jan.13044
10. Clark, M., Semple, M. J., Ivins, N., Mahoney, K., & Harding, K. (2017). National audit of pressure ulcers and incontinence-associated dermatitis in hospitals across Wales: A cross-sectional study [Article]. BMJ Open, 7(8). <https://doi.org/https://dx.doi.org/10.1136/bmjopen-2016-015616>
11. Gonzalez-Mendez, M. I., Lima-Serrano, M., Martin-Castano, C., Alonso-Araujo, I., & Lima-Rodriguez, J. S. (2017). Incidence and risk factors associated with the development of pressure ulcers in an intensive care unit [Article]. Journal of clinical nursing, 27(5-6), 1028-1037. <https://doi.org/https://dx.doi.org/10.1111/jocn.14091>
12. Eglseer, D., Hodl, M., & Lohrmann, C. (2019). Six Nursing Care Problems in Hospitals: A Cross-Sectional Study of Quality of Care. Journal of nursing care quality, 34(1), E8-E14. https://doi.org/https://dx.doi.org/10.1097
13. De Meyer, D., Verhaeghe, S., Van Hecke, A., & Beeckman, D. (2019). Knowledge of nurses and nursing assistants about pressure ulcer prevention: A survey in 16 Belgian hospitals using the PUKAT 2.0 tool [Article]. Journal of Tissue Viability, 28(2), 59-69. <https://doi.org/https://dx.doi.org/10.1016/j.jtv.2019.03.002>
14. Biz, C., Fantoni, I., Crepaldi, N., Zonta, F., Buffon, L., Corradin, M., Lissandron, A., & Ruggieri, P. (2019). Clinical practice and nursing management of pre-operative skin or skeletal traction for hip fractures in elderly patients: a cross-sectional three-institution study [Article]. International journal of orthopaedic and trauma nursing, 32(pp 32-40). <https://doi.org/https://dx.doi.org/10.1016/j.ijotn.2018.10.002>
15. Hödl, M., & Voithofer, C. (2019). [Pressure ulcer risk assessment and preventive measures in mobile / chairfast / bedfast hospital patients] [Multicenter Study]. Pflege, 32(4), 181-187. https://doi.org/https://dx.doi.org/10.1024/1012-5302/a000678 (Dekubitusrisikoeinschätzung und präventive Maßnahmen bei Krankenhauspatientinnen und -patienten: Gehen-Sitzen-Liegen)
16. Charalambous, C., Koulouri, A., Roupa, Z., Vasilopoulos, A., Kyriakou, M., & Vasiliou, M. (2019). Knowledge and attitudes of nurses in a major public hospital in Cyprus towards pressure ulcer prevention [Article]. Journal of Tissue Viability, 28(1), 40-45. <https://doi.org/https://dx.doi.org/10.1016/j.jtv.2018.10.005>
17. Lichterfeld-Kottner, A., Lahmann, N., & Kottner, J. (2020). Sex-specific differences in prevention and treatment of institutional-acquired pressure ulcers in hospitals and nursing homes. Journal of Tissue Viability, 29(3), 204-210. <https://doi.org/https://dx.doi.org/10.1016/j.jtv.2020.05.001>
18. Lopez-Franco, M. D., Parra-Anguita, L., Comino-Sanz, I. M., & Pancorbo-Hidalgo, P. L. (2020). Attitudes of Spanish nurses towards pressure injury prevention and psychometric characteristics of the spanish version of the ApuP instrument [Article]. International Journal of Environmental Research and Public Health, 17(22), 1-15. <https://doi.org/https://dx.doi.org/10.3390/ijerph17228543>
19. Gaspar, S., Collier, M., Marques, A., Ferreira, C., & Gaspar de Matos, M. (2020). Pressure ulcers: The challenge of monitoring in hospital context. Applied nursing research: ANR, 53(pp 151266). <https://doi.org/https://dx.doi.org/10.1016/j.apnr.2020.151266>
20. Strube-Lahmann, S., & Lahmann, N. A. (2021). Pressure ulcer prevention—Results of a multicentre cross-sectional survey on hospital infrastructures and processes in acute hospitals and accident and emergency departments. Journal of Evaluation in Clinical Practice, 27, 1361–1368. <https://doi.org/10.1111/jep.13571>
21. Eglseer, D., Osmancevic, S., Hoedl, M., Lohrmann, C., & Bauer, S. (2021). Improving the quality of nursing care in Austria: 10 years of success. Journal of nursing management, 29(2), 186-193. <https://doi.org/https://dx.doi.org/10.1111/jonm.13136>
22. Halasz, B. G., Beresova, A., Tkacova, L., Magurova, D., & Lizakova, L. (2021). Nurses' knowledge and attitudes towards prevention of pressure ulcers. International Journal of Environmental Research and Public Health, 18(4), 1-9. <https://doi.org/https://dx.doi.org/10.3390/ijerph18041705>
23. Källman, U., Hommel, A., Borgstedt Risberg, M., Gunningberg, L., Sving, E., & Bååth, C. (2022). Pressure ulcer prevalence and prevention interventions – A ten‐year nationwide survey in Sweden. International Wound Journal, 19(7), 1736-1747. <https://doi.org/10.1111/iwj.13779>
24. Parisod, H., Holopainen, A., Koivunen, M., Puukka, P., & Haavisto, E. (2022). Factors determining nurses' knowledge of evidence-based pressure ulcer prevention practices in Finland: a correlational cross-sectional study [Article]. Scandinavian journal of caring sciences, 36(1), 150-161. <https://doi.org/https://dx.doi.org/10.1111/scs.12972>
25. Haavisto, E., Stolt, M., Puukka, P., Korhonen, T., & Kielo-Viljamaa, E. (2021). Consistent practices in pressure ulcer prevention based on international care guidelines: A cross-sectional study. International Wound Journal, 19(5), 1141-1157. <https://doi.org/https://dx.doi.org/10.1111/iwj.13710>
26. Stephenson, J., Obe, J. F., Parfitt, G., & Ousey, K. (2021). National audit of pressure ulcer prevalence in England: A cross sectional study [Article]. Wounds UK, 17(4), 45-55.
27. Avgerinou, I., Kalemikerakis, I., Vasilopoulos, G., Kelesi, M., Polikandrioti, M., Petsios, K., & Dousis, E. (2022). Intensive Care Nurses’ Knowledge, Practice and Attitudes Related to Pressure Ulcer Prevention: A Single Tertiary Center in Greece. International Journal of Caring Sciences, 15(2), 780.
28. Cukljek, S., Rezic, S., Ficko, S. L., Hosnjak, A. M., Smrekar, M., & Ljubas, A. (2022). Croatian nurses' and nursing students' knowledge about pressure injury prevention [Article]. Journal of Tissue Viability, 31(3), 453-458. <https://doi.org/https://dx.doi.org/10.1016/j.jtv.2022.04.008>
29. Andersson, J., Imberg, S., & Rosengren, K. (2022). Documentation of pressure ulcers in medical records at an internal medicine ward in university hospital in western Sweden [Article]. Nursing open, 10(3), 1794-1802. <https://doi.org/https://dx.doi.org/10.1002/nop2.1439>
30. Eirinidou, P., Gerogianni, G., Vasilopoulos, G., Kalemikerakis, I., Kalogianni, A., Kaba, E., Fasoi, G., Zartaloudi, A., & Kelesi, M. (2023). Nurses' Knowledge Concerning Prevention and Treatment of Pressure Ulcers [Chapter]. Advances in Experimental Medicine and Biology. <https://doi.org/https://dx.doi.org/10.1007/978-3-031-31986-0_58>
31. Vivero, A. F., Areosa, L. A., & Prieto, J. M. R. (2023). Barriers and attitudes to prevent the adverse effect of pressure ulcers in a trauma unit [Article]. Gerokomos, 34(1), 46-52.
32. Tervo-Heikkinen, T., Heikkila, A., Koivunen, M., Kortteisto, T., Peltokoski, J., Salmela, S., Sankelo, M., Ylitormanen, T., & Junttila, K. (2023). Nursing interventions in preventing pressure injuries in acute inpatient care: a cross-sectional national study. BMC Nursing, 22(1), 198. <https://doi.org/https://dx.doi.org/10.1186/s12912-023-01369-8>
33. Bjurbo, C., Wetzer, E., Thunborg, D., Zhang, L., & Hultin, L. (2024). Knowledge and attitudes regarding pressure injuries among assistant nurses in a clinical context [Article]. International Wound Journal, 21(7). <https://doi.org/https://dx.doi.org/10.1111/iwj.14950>
34. Celik, B., Turhan Damar, H., Savsar, A., Ferraiuolo, F., Repustic, M., & Ogce Aktas, F. (2024). Investigation of related factors of operating room nurses' attitudes and awareness towards surgery-related pressure injury prevention in Turkey, Croatia, and Italy [Article]. Journal of Tissue Viability, 33(3), 418-424. https://doi.org/https://dx.doi.org/10.1016/j.jtv.2024.05.003

Table 3 Mixed Methods

| **No.** | **Author (Year)** | **Country** | **Setting** | **Objective** | **Method** | **Sample** | **Main variables** | **Main Results** |
| --- | --- | --- | --- | --- | --- | --- | --- | --- |
|  | Balzer. et al.  (2014) | - Germany | - Hospital - Traumatological wards (n=2) | - Exploring nurses’ clinical judgement about pressure ulcers without using risk assessment tool | - Qualitative study: - Interviews, semi-structured - Case vignettes (n=8) from quantitative data | - Purposively selected Nurses (n=16, 8 per ward) | - Braden Scale - Care Dependency Scale (CDS) - Mobility support needs - Continence status - Nutritional status (NRS 2000) - Admission diagnosis (e.g. hip fracture) - Co-morbidities - Age, gender, BMI - Interviews: - High motivation - Desired social participation - Willingness to cooperate - Expected good compliance - Coping with risk - - Self-care behaviour | - Nurses relied most on factors reflecting exposure to pressure and care dependency (e.g. mobility limitations, continence status, hip injuries). These showed the strongest quantitative correlations with risk judgement - Nurses also factored in protective characteristics like patient motivation, compliance, and self-care ability, which are not captured by formal scales - Triangulation showed strong alignment between clinical judgement and structured tools (Braden, CDS), but also revealed how nurses used accurate reasoning that included psychosocial and behavioural factors. - Clinical judgement went beyond the scope of standardized tools, that means, nurses’ clinical judgment included behavioral, motivational, and contextual factors that standardized tools and instrument ignore |
|  |  |  |  |  | - Quantitative part:   Quasi-experimental trial | - Patients (n=106) - Age: ≥18 years - no category ≥2 PUs at admission. - ≥5 days stay |  |  |
|  | Worsley et al.  (2017) | - UK | - Hospital | - Assessment of attitudes and knowledge towards PU prevention - Identifying barriers and facilitators for PU prevention and treatment | - Focus group, semi-structured - Content analysis | - Physiotherapists (n= 5) - Occupational therapists (n=4) - Age: 27-57 years - Female: n=7 | - Attitudes assessment: 13 items - Knowledge assessment: 26 items, 6 key themes | - Barriers: - Therapists feel not involved in PU prevention strategies of the hospital - Lack of communication with nursing staff - Facilitators: - Prevention is part of role and responsibility of therapy staff |
|  |  |  |  |  | - Survey research, 2 different questionnaires |  |  |  |
|  | Coleman et al.  (2024) | - UK | - Hospitals   (n=4) | - Observing clinical judgement and decision making about care planning and delivery | - Realist evaluation |  | PURPOSE-T usage  Nurse experience  Documentation  Repositioning schedules  PU presence | - PURPOSE-T used differently by experience level - Documentation and blame culture influenced care. (A “blame culture” was described, where staff feared being personally held responsible if a pressure ulcer developed, despite official messages of a “no blame culture.”   This made the use of PURPOSE-T as a protective mechanism, helping staff “prove” they followed the correct process.)   - One-size-fits-all approach prevailed (the same care is given to everyone, even when not ideal) - Competing priorities like falls prevention   (other safety tasks like preventing falls sometimes get more attention).   - Basic equipment is used for everyone, but not always adjusted based on the patient’s situation. |
|  |  |  |  |  | - Ethnographic observation 75 h - interview, semi-structured - policy review - Patient record review | Purpose sampling of:   - 72 patients (observed) - 15 patient (records reviewed) - 16 staff (interviewed) |  |  |
|  | Greenwood et al. (2024) | - UK | - Hospital - Orthopedic wards (n=3) | Exploring how and why heel offloading devices are used and reasoning behind their use in population at high PU risk on heels | - Ethnographic study with program theory - Short interviews post observations with staff members (n=19) - Depth interviews with ward managers (n=3) | - Observations (n=12) - Patients (n=32) - Age, mean 73.9 years - Female, n=23 | - Standard care included risk assessment within 6 hours after admission, electric profiling bed, high specification foam mattress and armchair | - Leadership of Tissue Viability Nurse and Ward manager are essential for successful use of devices, e.g. a clear protocol, which device should be uses for which patient type - Offloading and constant low-pressure devices are used in practice - Although there is a lack of evidence, low-pressure devices are perceived as beneficial for prevention and offloading devices are seen better for treatment of heel PUs |

1. Balzer, K., Kremer, L., Junghans, A., Halfens, R. J., Dassen, T., & Kottner, J. (2014). What patient characteristics guide nurses' clinical judgement on pressure ulcer risk? A mixed methods study. International journal of nursing studies, 51(5), 703-716. <https://doi.org/https://dx.doi.org/10.1016/j.ijnurstu.2013.09.005>
2. Worsley, P. R., Clarkson, P., Bader, D. L., & Schoonhoven, L. (2017). Identifying barriers and facilitators to participation in pressure ulcer prevention in allied healthcare professionals: a mixed methods evaluation [Article]. Physiotherapy, 103(3), 304-310. <https://doi.org/https://dx.doi.org/10.1016/j.physio.2016.02.005>
3. Coleman, S., Greenhalgh, J., Schoonhoven, L., Twiddy, M., & Nixon, J. (2024). Using PURPOSE-T in clinical practice: A realist evaluation [Article]. Journal of Tissue Viability, 33(4), 672-680. <https://doi.org/https://dx.doi.org/10.1016/j.jtv.2024.06.014>
4. Greenwood, C., Nixon, J., Nelson, E. A., McGinnis, E., & Randell, R. (2024). A realist evaluation of devices used for the prevention of heel pressure ulcers: An ethnographic study of clinical practice [Article]. Applied nursing research: ANR, 76(pp 151785). <https://doi.org/https://dx.doi.org/10.1016/j.apnr.2024.151785>
